# Proximity-specific ribosome profiling reveals the logic of localized mitochondrial translation

**DOI:** 10.1016/j.cell.2025.08.002

**Published:** 2025-08-27

**Authors:** Jingchuan Luo, Stuti Khandwala, Jingjie Hu, Song-Yi Lee, Kelsey L. Hickey, Zebulon G. Levine, J. Wade Harper, Alice Y. Ting, Jonathan S. Weissman

**Affiliations:** 1 Whitehead Institute for Biomedical Research, Cambridge, MA, USA; 2 Department of Genetics, Stanford University, Stanford, CA, USA; 3 Department of Genetics, Biology, and by courtesy, Chemistry, Stanford University School of Medicine, Stanford, CA 94305, USA; Chan Zuckerberg Biohub – San Francisco, San Francisco, CA 94158, USA; 4 Department of Biology, Massachusetts Institute of Technology, Cambridge, MA, USA; 5 David H. Koch Institute for Integrative Cancer Research, Massachusetts Institute of Technology; Cambridge, MA 02142, USA; 6 Howard Hughes Medical Institute, Massachusetts Institute of Technology, Cambridge, MA, USA; 7 Department of Cell Biology, Blavatnik Institute, Harvard Medical School, Boston, MA 02115, USA; 8 These authors contributed equally.; 9 Current affiliation: Department of New Biology, DGIST, Daegu 42988, Republic of Korea; New Biology Research Center, DGIST, Daegu 42988, Republic of Korea

**Keywords:** localized translation, outer mitochondrial membrane, cis-element analysis, oxidative phosphorylation (OXPHOS), AKAP1, mitochondrial bipartite targeting signal, cotranslational targeting, translation-independent mRNA targeting

## Abstract

Localized translation broadly enables spatiotemporal control of gene expression. Here we present LOCL-TL (LOV-domain-Controlled Ligase for Translation Localization), an optogenetic approach for monitoring translation with codon resolution at any defined subcellular location under physiological conditions. Application of LOCL-TL to mitochondrially localized translation revealed that ~20% of human nuclear-encoded mitochondrial genes are translated on the outer mitochondrial membrane (OMM). Mitochondrially-translated messages form two classes distinguished by encoded protein length, recruitment mechanism, and cellular function. An evolutionarily ancient mechanism allows nascent chains to drive cotranslational recruitment of long proteins via an unanticipated bipartite targeting signal. Conversely, mRNAs of short proteins, especially eukaryotic origin electron transport chain (ETC) components, are specifically recruited by the OMM protein A-Kinase Anchoring Protein 1 (AKAP1), in a translation-independent manner that depends on mRNA splicing. AKAP1 loss lowers ETC levels. LOCL-TL thus reveals a hierarchical strategy that enables preferential translation of a subset of proteins on the OMM.

## In brief

Development of LOCL-TL (LOV-domain-Controlled Ligase for Translation Localization), an optogenetic approach for monitoring translation with codon resolution at any defined subcellular location under physiological conditions, reveals two mechanistically and functionally distinct strategies for mitochondrially localized translation.

## Graphical Abstract

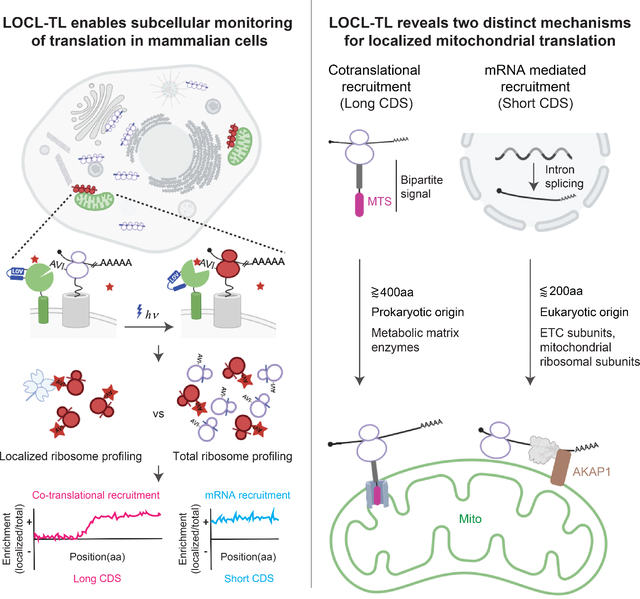

## INTRODUCTION

Mitochondria originated from α-proteobacteria via endosymbiosis, roughly 1.5 billion years ago^1^. The vast majority of mitochondrial genes have since migrated to the nuclear genome, although a handful of genes (13 in humans) remain encoded in the mitochondrial genome^1,2^. Nuclear-encoded mitochondrial proteins are synthesized in the cytoplasm and imported into the mitochondria, and the production of nuclear and mitochondrial encoded proteins is coordinated through a variety of processes^1,3,4^. Historically, post-translational insertion was considered the dominant mechanism for mitochondrial protein import^5^. Nevertheless, recent observations that the translation machinery and a subset of mRNA transcripts localize near the outer mitochondrial membrane (OMM) indicate that a subset of proteins may be cotranslationally imported^6–13^. In yeast, localized translation may prevent undesired misfolding or accumulation of mitochondrial proteins in unintended locations^6,10^. Localized translation could promote rapid changes in individual mitochondrial proteomes or facilitate complex assembly. However, the physiological prevalence and significance of mitochondrially localized translation, especially in higher eukaryotes, remains unclear.

This question has been most thoroughly examined in the budding yeast *Saccharomyces cerevisiae*^6,7,9,10,13,14^. Electron cryotomography revealed the presence of ribosomes on the surface of yeast mitochondria^7^. The mitochondrial outer membrane protein OM14 has been implicated in recruiting nascent polypeptide-associated complex (NAC) and cytosolic ribosomes to the mitochondrial surface^14^. Additionally, Puf3, a Pumilio family RNA binding protein, binds to the 3′ UTR of a subset of locally translated transcripts and targets them to the OMM^9^. The most direct evidence for localized translation of proteins destined for mitochondria comes from the application of proximity-specific ribosome profiling, which precisely monitors protein synthesis at subcellular locations^6,15^. This technology specifically labels locally-translating ribosomes that are in proximity to a biotin ligase - BirA. By targeting BirA to a specific subcellular location, such as the yeast outer mitochondrial membrane, a controlled biotin pulse allows BirA to label nearby ribosomal subunits that are fused to the biotin ligase peptide substrate (Avi-tag). The biotinylated ribosome can then be affinity purified and the position of the mRNA it is decoding revealed by ribosome profiling (deep-sequencing of ribosome protected fragments)^16^. This proximity-specific ribosome profiling approach revealed that an important subset of nuclear-encoded mitochondrial mRNAs, most of which encode inner membrane proteins, are locally translated at the yeast OMM.

In mammalian cells, however, localized translation of mitochondrial proteins is far less explored. Mitochondrially localized translation in humans is likely to be mechanistically distinct from that in yeast since human homologs of OM14 and Puf3 have not been identified. A few mRNA binding proteins have been associated with recruiting mRNA to the OMM but their molecular mechanisms and substrate range are largely unresolved^12,17–19^. Recent application of RNA proximity labeling methods, such as APEX-seq^8,20^, reveals that a subset of mitochondrial and cytosolic transcripts is in proximity to the OMM, with some of their recruitment being dependent on ribosomal binding and translation. However, this approach does not directly examine translation or determine when the translational machinery is engaged. The original proximity-specific ribosome profiling method could in principle resolve these questions^6,15,21^, but is challenging to apply in mammalian cells due to the need to deplete the essential vitamin biotin in order to prevent premature labeling of ribosomes.

Here, we used an optogenetic approach to develop a general tool, termed LOV-domain Controlled Ligase for Translation Localization (LOCL-TL), to enable proximity-specific ribosome profiling under normal growth conditions. This allows us to define the nature and mechanism of local translation for mitochondrial proteins and, more generally, provides an approach for monitoring translation in any subcellular location in mammalian cells under physiological conditions.

## RESULTS

### Establishment of LOCL-TL: an optogenetic approach for proximity-specific ribosome profiling

**Figure 1.**
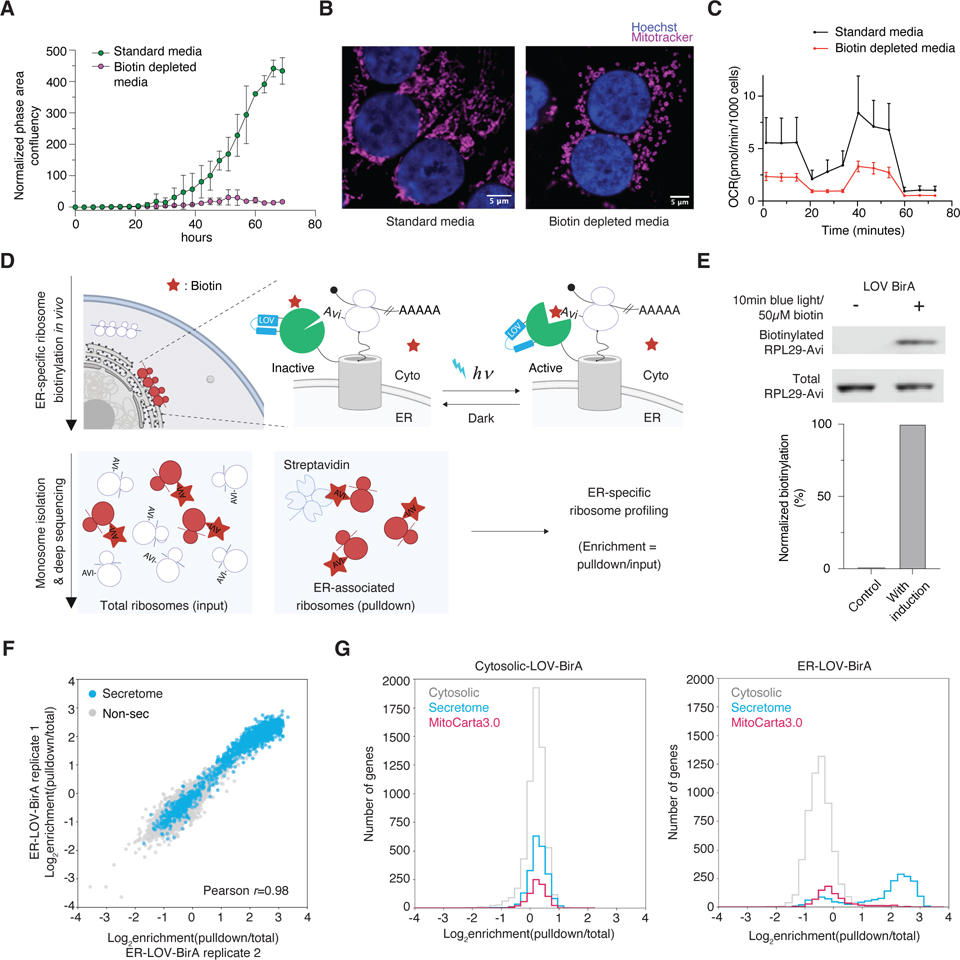
Optogenetically controlled proximity-specific ribosome profiling (LOCL-TL) (A) Incucyte growth assays of HEK293T cells in various media. (B) Confocal images of HEK293T cells showing mitochondrial morphology (MitoTracker, magenta) and nuclei (Hoechst, blue) after two weeks in different media. Scale bar represents 5 μm. (C) Seahorse assay of HEK293T cells grown in various media. (D) Schematic of LOCL-TL. With a short pulse of blue light, LOV-BirA-Sec61β (blue - green - gray) at the ER membrane is activated, biotinylating the Avi-tag ribosomal subunits in proximity (red). Monosomes are generated via MNase digestion, and footprints from biotinylated monosomes are enriched with streptavidin beads for ribosome profiling. (E) Western blot and quantification of biotinylated RPL29-Avi (streptavidin) vs. total RPL29-Avi (HA antibody) upon induction. (F) Enrichment of representative ER specific LOCL-TL replicates for secretome (blue) and other (gray) gene categories. (G) Histograms of log_2_ enrichment for secretory (blue), MitoCarta3.0 (red) and other cytosolic (gray) genes in HEK293T cells expressing endogenously tagged RPL29-Avi fusion proteins and cytosolic-LOV-BirA or ER-LOV-BirA. See also Figures S1 and S2.

Proximity-specific ribosome profiling critically depends on the ability to precisely control the location and timing of ribosome labeling. Without temporal control, labeled ribosomes rapidly recycle back to the general cytosolic pool, losing spatial information. In standard media containing ample biotin, BirA continuously labels its substrate, a short peptide sequence termed an Avi tag (Figure S1A). In our original implementation, temporal control was achieved by growing yeast cells in biotin-depleted media and initiating labeling with biotin addition. While biotin depletion was reasonably well-tolerated in yeast, for mammalian cells this leads to mitochondrial fragmentation, mitochondrial dysfunction and strong cell growth defects (Figures 1A, 1B & 1C). To circumvent this, we sought to use blue light as an alternative way to temporally regulate biotin ligase activity (Figure 1D). We built on our recently described LOV-Turbo^22^, which allows for optogenetic control of a promiscuous biotin ligase. Specifically, we engineered a light regulated biotin ligase (LOV-BirA) by incorporating the LOV photosensory domain, a 16kDa flavin-containing protein from the *Avena sativa* oat plant, into BirA. Unlike promiscuous biotinylation enzymes, LOV-BirA is a site-specific ligase that requires direct contact with Avi-tagged ribosomes to biotinylate the Avi tag^23^. This specificity arises because LOV-BirA transfers biotin only from biotin-AMP onto Avi-tagged proteins that dock directly into its active site. Optimization of the LOV-BirA fusion allowed us to achieve tight and rapid light control (Figures S1B and S1C): prior to exposure to blue light, LOV-BirA is inactive and shows minimal background biotinylation, even in media with normal biotin levels (Figures 1E and S1A). Exposure to blue light leads to rapid biotinylation of Avi-tagged ribosomes.

We benchmarked the LOCL-TL approach by monitoring translation at the surface of the endoplasmic reticulum (ER) which has been extensively studied as the site of cotranslational translocation of secreted proteins^15,24,25^. LOV-BirA was targeted to the ER membrane by fusing it with Sec61β, a tail-anchored ER membrane protein present in Sec61 translocons. The acceptor peptide - Avi tag was fused to the N terminus of ribosomal subunit RPL10A (Figure 1D). After briefly treating cells with the translation elongation inhibitor cycloheximide (CHX), we activated LOV-BirA with a 10-minute pulse of blue light, biotinylating proximal ribosomes. We then treated labeled ribosomes with micrococcal nuclease to generate monosomes, followed by affinity purification of biotinylated ribosomes and sequencing of the nuclease-protected mRNA ribosome footprints. Enrichment scores for each gene were calculated by normalizing the pulldown to the total input (Figure 1D). Secretory genes were highly enriched using ER-localized LOV-BirA, but not with the cytosolic version (Figure S1D). Our results are highly correlated with results obtained using the previous proximity-specific ribosome profiling approach requiring biotin depletion (Figure S2A, Pearson *r* = 0.95, Supplemental Table 1). Together these findings establish the ability of LOCL-TL to accurately monitor localized translation under normal physiological growth conditions.

We next identified ribosomal subunits that could be tagged at their endogenous loci without impacting function. We picked four subunits: RPL10A, RPL13, RPL29 and RPL36 (Figure S2B), based on the following criteria: (i) surface exposure of the terminus; (ii) avoidance of the interface of the large and small ribosomes; (iii) appropriate distance from the Sec61 translocon to avoid interfering with ribosomal binding while not being too distal for efficient labeling^26^. We chose endogenously tagged RPL29 cell lines for subsequent experiments because LOV-BirA effectively biotinylated Avi-tagged RPL29 (Figure S2C), the overall translation activity is not detectably impacted by Avi-tagged RPL29 (Figure S2D) and homozygous RPL29 knock-in cell lines grew comparably to wild-type HEK293T cells (Figure S2E). Additionally, minimal post-lysis labeling occurred even when light was present during processing. (Figure S2G, see methods).

We performed LOCL-TL with engineered RPL29-Avi cell lines expressing ER-localized LOV-BirA. The two replicates were highly correlated (Figure 1F, Pearson *r* = 0.98, Supplemental Table 1). Secretory proteins were highly enriched in the dataset, while negative controls (mitochondrial and cytosolic proteins) were not (Figure 1G, Supplemental Table 1). As an additional control, we used cytosolic LOV-BirA and observed that secretory proteins were no longer enriched (Figure 1G). The results from RPL29-Avi cell lines were also highly correlated with those from Avi-RPL10A cells (Figure S2F, Pearson *r* = 0.95, Supplemental Table 1). We therefore focused our study on endogenously tagged RPL29-Avi cell lines, unless otherwise specified.

### The landscape of mitochondrially localized translatome in mammalian cells

**Figure 2.**
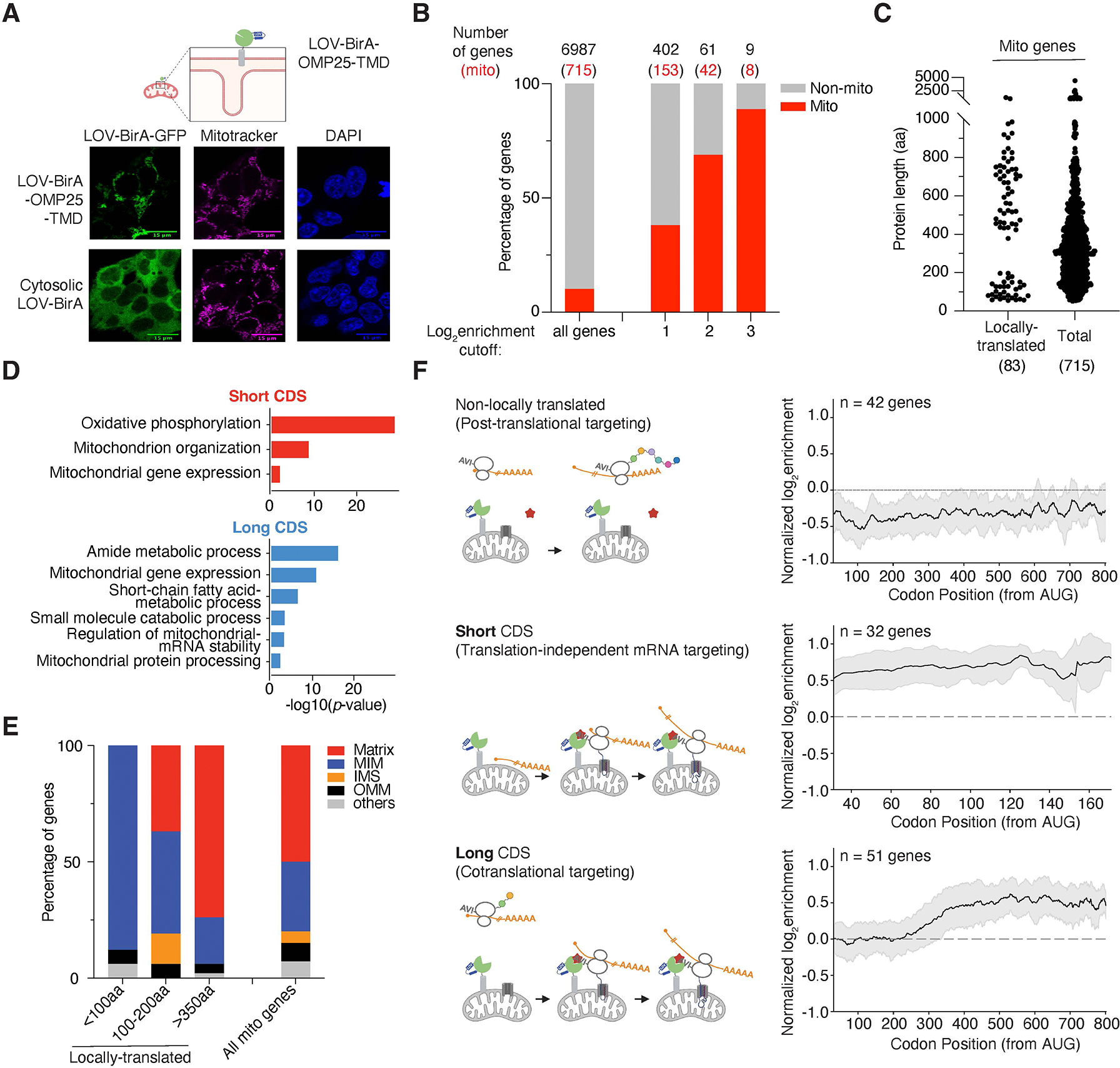
Mitochondrial-specific LOCL-TL reveals two distinct classes of mitochondrially localized translation (A) Confocal images of HEK293T cells expressing LOV-BirA-GFP fusion proteins with mitochondria (MitoTracker, magenta) and nuclei (DAPI, blue). A cartoon illustrates the LOV-BirA-GFP-OMP25-TMD fusion protein localized to the OMM, with LOV-BirA facing the cytosol. Scale bar represents 15 μm. (B) Bar graph showing the percentage of mitochondrial (red) vs. non-mitochondrial genes (gray) whose enrichments exceed the indicated log_2_ enrichment threshold. The total gene count per group is displayed above each bar, with mitochondrial gene counts in red. (C) Dot plot comparing protein lengths of locally translated genes and all mitochondrial genes, with the number of mitochondrial genes in parentheses. (D) GO term analysis^33^ of locally translated short and long CDS groups from (C). (E) Bar graph showing the percentage of genes for different mitochondrial sub-compartments, categorized by protein length cut-offs, comparing locally-translated genes to all mitochondrial genes (input control). (F) Models illustrating different translation dynamics (left) and corresponding metagene plots (right) of localized translation at codon-level resolution. See also Figure S3.

To study localized translation at the OMM, we fused LOV-BirA with various OMM proteins: TOM5, TOM70 and the transmembrane domain (TMD) of OMP25. We chose OMP25 TMD rather than the full-length protein because (1) the TMD domain effectively targets fusion proteins to the OMM^27^, and (2) we aimed to minimize the impact of overexpressing functional OMP25^28^. GFP was included in each fusion for imaging subcellular locations, and the fusion proteins are expressed under the EF1α promoter and stably integrated into HEK293T cells via lentiviral transduction. All fusion proteins were properly expressed and appeared at the expected size by western blot analysis (Figure S3A). Fluorescent confocal images confirmed correct localization of fusion proteins to the mitochondria (Figures 2A and S3B). Among the different mito-LOV-BirAs, the OMP25 fusion showed the highest enrichment of nuclear-encoded mitochondrial proteins as defined by MitoCarta3.0^29^ (Figures 2B and S3D). Therefore, we selected LOV-BirA-OMP25-TMD cell lines for the subsequent studies.

Using this engineered system, we generated a systematic map of mitochondrially localized translation in mammalian cells. LOCL-TL revealed that ~20% of nuclear-encoded mitochondrial proteins are preferentially translated on the OMM but it remains possible that even for this subset there is a pool that is translated in the cytosol. Biological replicates of enrichment of translation on the OMM for each transcript showed substantial agreement (Figure S3C, Pearson *r* = 0.88, Supplemental Table 2). Furthermore, the set of transcripts showing preferential translation on the OMM was highly enriched for genes encoding mitochondrial proteins (Figure 2B, Supplemental Table 2). Nonetheless, a prominent subset of transcripts encoding non-mitochondrial proteins, were found to be preferentially translated on the OMM. The expression level of this subset did not correlate with enrichment scores, arguing that they were not artifacts of abundance (Figure S3F, Pearson *r* = 0.32). We compared our dataset to a previous APEX-seq dataset that detected transcripts near the OMM^8^ and found that ~85% of transcripts encoding cytosolic proteins enriched in our dataset were also enriched in the APEX-seq dataset (Figure S3G, Pearson *r* = 0.67). By contrast, a large fraction of the transcripts encoding cytosolic proteins that were found to be enriched on the OMM by APEX-seq show minimal enrichment of OMM translation in our data, suggesting that the enriched non-mitochondrial genes in our LOCL-TL data are genuine signals, even though most of them have not been linked to mitochondria. The vast majority (99%) of nuclear-encoded mitochondrial genes showing enriched translation on the OMM in our dataset were also enriched in the APEX-seq data (Figure S3H, Pearson *r* = 0.62). Together these observations argue for the high specificity of the LOCL-TL approach. By contrast, ~25% of transcripts found to be enriched on the OMM by APEX-seq are preferentially translated on the OMM by the LOCL-TL approach (Figure S3H). This discrepancy could be partially due to our method directly detecting actively translating transcripts, while APEX-seq detects the total pool of nearby transcripts, both translating and non-translating. Additionally, APEX labeling is via a released radical intermediate which has a longer labeling radius than the contact-based labeling mechanism used here^8,20^ and thus will have lower spatial resolution (Figure S3E). Similarly, the approach based on TurboID^30,31^ we recently described for labeling ribosomes based on location has a more extended labeling radius making it better suited for applications, such as the study of translation in postsynaptic compartments^32^, where one wishes to label an entire subcellular compartment.

### Two classes of proteins translated at the OMM

Remarkably, a plot of protein lengths of locally translated genes revealed two distinct populations: short coding sequences (CDSes) (<200aa) and long CDSes (>400aa) (Figure 2C, Supplemental Table 2); this bimodal distribution was markedly different from the overall size distribution of nuclear-encoded mitochondrial proteins. GO term analysis^33^ for the short CDS group indicated that they were highly enriched for electron transport chain (ETC) subunits and mitochondrial ribosomal subunits, whereas the long CDS group was enriched for tRNA synthetases and genes from various metabolic pathways (Figure 2D). Consistently, long CDS proteins were mostly mitochondrial matrix-localized proteins, while short CDS proteins, especially the ones less than 100aa, were predominantly localized to the inner membrane where the ETC is found (Figure 2E).

To investigate potential differences in the timing of translation localization to the OMM, we generated metagene plots of OMM enrichment as a function of distance from the start codon taking advantage of the codon-resolution of ribosome profiling^16^. For genes that showed no overall enrichment on the OMM (non-locally translated), the enrichment value was low throughout the transcript (Figure 2F, Supplemental Table 2). For locally translated short CDS genes, local translation starts from the beginning of the transcript and is consistent throughout the transcript’s length, suggesting that transcripts are targeted to the OMM in a translation-independent manner. By contrast, for long CDS genes translated at the OMM, the first ~250 aa are not enriched, indicating that protein synthesis begins in the cytosol and the ribosome/nascent chain complex is recruited to the OMM over the next ~150 aa to finish translation. These findings underscore the value of the ability to make codon-level measurement of cotranslational recruitment in mammalian mitochondria now enabled by the LOCL-TL approach.

### Development of a rapid intact mRNA-based readout for LOCL-TL

**Figure 3.**
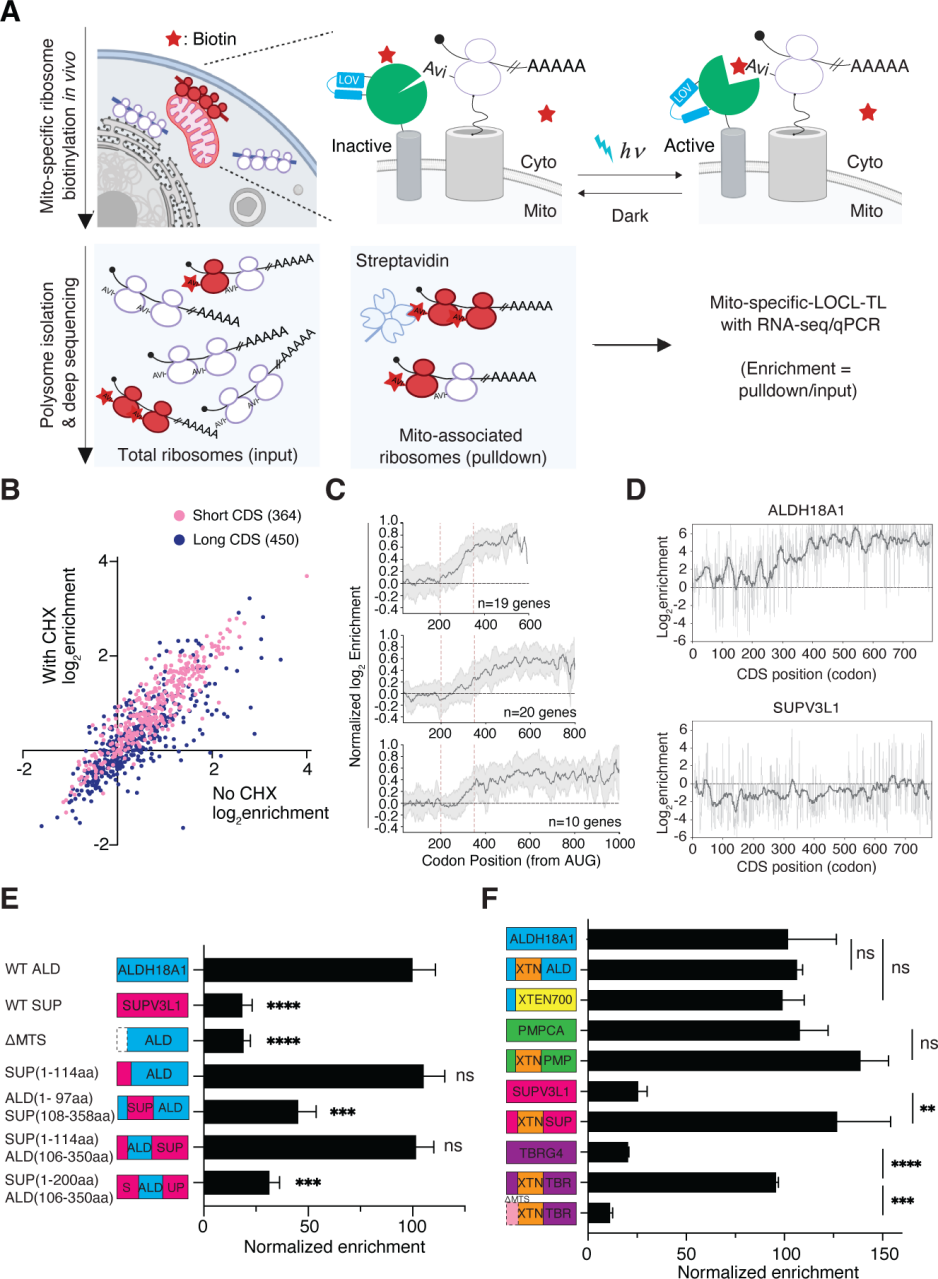
Localized translation of long CDSes is mediated by a bipartite signal sequence. (A) Alternative LOCL-TL schematic with RNA-seq/qPCR readout. Transcripts actively translated by biotinylated ribosomes are enriched with streptavidin beads for subsequent RNA-seq or qPCR quantification. Colors match Fig. 1D. (B) Scatter plot of log_2_ enrichment from mitochondrial-specific LOCL-TL profiling by RNA-seq, with or without cycloheximide. Short CDSes are pink; Long CDSes are blue. The number of mitochondrial genes is indicated in parentheses. (C) Metagene plots of locally translated long CDSes (400–600 aa, 600–800 aa and 800–1000 aa) from mitochondrial-specific LOCL-TL profiling by ribosome profiling. Red dotted lines indicate the 200 aa and 350 aa positions. (D) Codon enrichment plots for locally translated ALDH18A1 and non-locally translated SUPV3L1 from mitochondrial-specific LOCL-TL profiling by ribosome profiling. (E) Cis-element analysis using chimeric long CDS reporters, measured by qPCR (N=3). Normalization details are in the Star Methods. *p*-values were generated by comparison to the positive reporter (top). ALD: ALDH18A1, SUP: SUPV3L1. (F) Additional cis-element analysis with chimeric long CDS reporters, measured by qPCR (N=3). XTN: XTEN250, PMP: PMPCA, SUP: SUPV3L1, TBR: TBRG4. Statistical significance was determined by two-tailed unpaired t-test. *p*-value > 0.05 (ns), *p*-value ≤ 0.05 (*), *p*-value ≤ 0.01 (**), *p*-value ≤ 0.001 (***), *p*-value ≤ 0.0001 (****). See also Figures S3 and S4.

To facilitate the mechanistic analysis of factors driving local translation, we developed an orthogonal intact mRNA-based readout of LOCL-TL (Figure 3A). Unlike the ribosome profiling readout, here we do not digest the cell lysate with nuclease prior to isolating biotinylated ribosomes. Instead, we use streptavidin beads to directly pulldown whole transcripts engaged by labeled ribosomes and purify them for RNA-seq or qPCR analyses. The direct pull-down approach enables more rapid iteration because it obviates the need to make and sequence ribosome footprint libraries, which is especially advantageous when one wants to use qPCR to focus on a limited set of genes. Notably, in the intact mRNA strategy, a single labeled ribosome is sufficient for pulldown of an entire transcript. Despite this difference, the enrichment as measured using pulldown data from the OMM correlates well with our ribosome profiling data (Figure S3J, Pearson *r* = 0.74, Supplemental Table 2). In addition to simplifying the experimental protocol, the intact mRNA pulldown strategy requires ~40x less starting material, albeit at the expense of losing the single codon resolution.

We used the intact mRNA pulldown strategy to evaluate whether local translation on the OMM was a consequence of CHX treatment. Cycloheximide inhibition allows one to precisely define the point in translation when ribosomes are recruited but also provides more time for recruitment of the mitochondrial targeting sequence (MTS) to the import machinery. However, when we compared enrichment data with and without CHX treatment (Figures 3B and S3I, Supplemental Table 2), we did not see a consistent decrease in the mitochondrial enrichment for either short or long CDSes in the absence of CHX; thus, recruitment of both classes of substrates occurs under conditions of ongoing translation.

### A bipartite protein targeting signal drives cotranslational recruitment of long CDSes

Metagene analysis of the full set of long CDS transcripts shows that on average cotranslational recruitment commences between amino acids 250–400 (Figure 2F). We explored whether the positioning of cotranslational recruitment was consistent across genes or instead reflected an average position among a range of possible recruitment sites. We separated the long CDS transcripts into three groups based on length (400–600 aa, 600–800 aa and 800–1000 aa) and assessed the metagene plots for each group. The transition region was in a similar position relative to the N-terminus regardless of protein length (Figure 3C). Moreover, this transition can be observed for individual transcripts but was completely absent from long transcripts that are not found to be on average locally translated on the OMM (e.g. SUPV3L1, Figure 3D). Thus, it is the distance from the N-terminus that dictates when a message is recruited. Nonetheless, individual transcripts have different transitions, with some occurring later than 250 amino acids (e.g., GFM2 and MIPEP, Figure S4A).

We next built a series of chimeric reporters to define the cis-elements that drive cotranslational recruitment of long CDS transcripts. The reporters were introduced by a lentivirus and the degree of mitochondrially localized translation enrichment was assessed using the intact mRNA pulldown assay with a qPCR readout. We chose the mitochondrial matrix protein Aldehyde dehydrogenase 18 family member A1 (ALDH18A1, 795 aa), one of the most strongly enriched genes (Fold enrichment=17.95±0.02) in our ribosome profiling dataset, as the starting point for this dissection and the matrix protein Suv3 like RNA helicase (SUPV3L1, 786 aa), with similar length but minimal locally translated (Fold enrichment=0.85±0.03), as a negative control (Figure 3D). Replacing the protein coding region of ALDH18A1 CDS with SUPV3L1 CDS eliminated localized translation (Figure 3E). Replacing the first 105 amino acids containing the predicted MTS^34^ of ALDH18A1 with the first 114 aa of SUPV3L1, which includes a canonical MTS, did not affect localized translation, indicating that a specialized MTS is not required for localized translation. However, a functioning MTS is required since deleting the predicted MTS from ALDH18A1 completely abolished localized translation (see also below).

To further define regions within the coding sequence that are important for localized translation, we swapped the region between amino acid 115–350 of SUPV3L1 with the corresponding region from ALDH18A1 and found that this was sufficient to drive localized translation of the fusion protein (Figure 3E). By contrast, the opposite swap abolished localized translation of ALDH18A1. Finer scale analysis revealed that residues 200–350 of ALDH18A1 did not support nascent chain recruitment, whereas ALDH18A1 residues 106–250 largely did (Figure S4B). Thus, recruitment of the translating protein is driven by the protein coding region directly preceding the point at which the nascent chain begins to engage mitochondria. Interestingly, the insertion of 100 amino acids immediately downstream of the MTS from a post-translationally targeted protein (SUPV3L1 residues 100–200) is able to prevent the cotranslational targeting of a protein derived from ALDH18A1 that would otherwise do so (Figure 3E). This argues for the presence of a dominant inhibitory mechanism in post-translational proteins that prevents translocation before translation is completed.

### Exploration of the inhibitory sequences driving post-translational translocation

To further investigate the inhibitory mechanism in post-translational proteins, we replaced this critical region with unstructured peptides (e.g., XTEN linkers^35^) or intrinsically disordered sequences derived from Elastin (ELN) and MUCIN 6 (MU) that were not derived from mitochondrial proteins. This modification converted post-translational proteins (e.g., SUPV3L1 and TBRG4) into cotranslationally targeted proteins in multiple settings (Figures 3F & S4C) in an MTS-dependent manner (Figures 3E & 3F). To determine the minimum requirement for the localized translation of long CDSes, we engineered a synthetic reporter containing the N-terminal 97-amino acid region from ALDH18A1, which includes a putative 64-amino acid MTS^34^, followed by XTEN700 (a low-complexity unstructured region). This construct is robustly targeted to the OMM during translation (Figure 3F). These experiments suggest that post-translationally targeted proteins contain specific inhibitory regions immediately downstream of the MTS. Additionally, we generated a variant with a frameshift in this critical region of the post-translational protein SUPV3L1, which also significantly enhanced its localized translation, suggesting that this inhibitory mechanism is mediated by the protein coding sequence, rather than through RNA (Figure S4C).

### mRNA and introns but not the protein coding sequence mediate local translation of short CDSes

**Figure 4.**
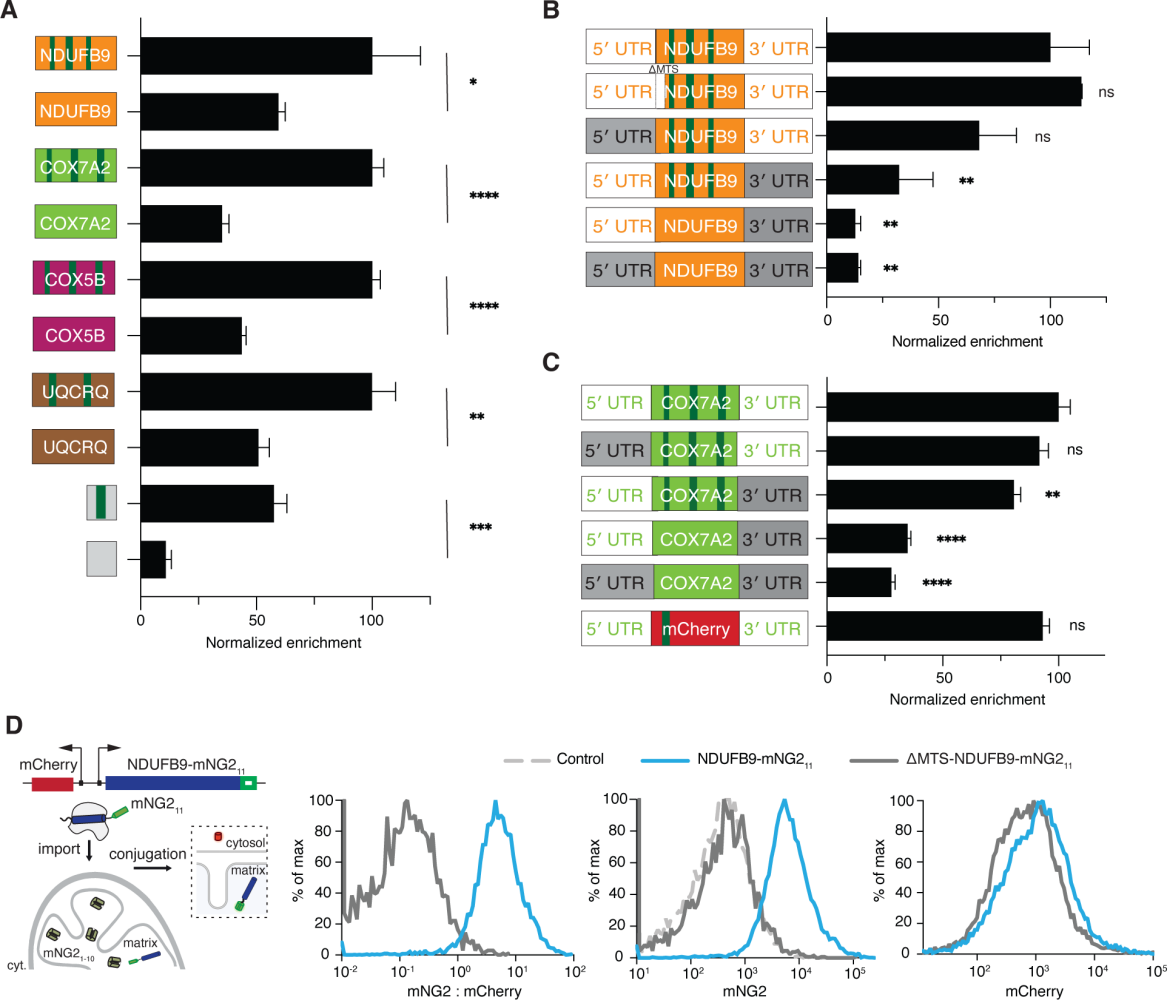
Localized translation of short CDSes is mediated by mRNA and introns, uncoupled from protein import. (A) Cis-element analysis of short CDS reporters. Endogenous and synthetic short CDS reporters were measured by qPCR (N=3). Green bars represent introns and the synthetic 49-amino acid short CDS reporter is light gray. Normalization details and the composition of the synthetic reporter are in the Star Methods. (B) Cis-element analysis of NDUFB9 reporters, measured by qPCR (N=3). Green bars represent introns and dark gray rectangles represent β-Actin 5′ or 3′ UTRs. *p*-values were generated by comparison to the positive NDUFB9 reporter (top). (C) Cis-element analysis of COX7A2 reporters. COX7A2 reporters, including a synthetic reporter with COX7A2 UTRs, a single intron and partial mCherry protein sequences, were measured by qPCR (N=3). Green bars represent introns and dark gray rectangles represent β-Actin 5′ or 3′ UTRs. *p*-values were generated by comparison to the positive COX7A2 reporter (top). (D) Split mNeonGreen2 reporter system for mitochondrial matrix import. A query protein fused to mNG2_11_ is expressed in a cell constitutively expressing mNG2_1–10_ in the matrix, along with a normalization marker (mCherry) driven by a separate promoter. Successful import into the matrix results in mNeonGreen2 fluorescence complementation. NDUFB9-mNG2_11_ reporters (with or without the MTS) were assessed in HEK293T cells expressing mNG2_1–10_ in the matrix. mNeonGreen2 fluorescence relative to the normalization marker mCherry was determined by flow cytometry and displayed as a histogram. Individual channels are also shown. The control is parental HEK293T cells expressing only mNG2_1–10_ in the matrix. Statistical significance was determined by two-tailed unpaired t-test. *p*-value > 0.05 (ns), *p*-value ≤ 0.05 (*), *p*-value ≤ 0.01 (**), *p*-value ≤ 0.001 (***), *p*-value ≤ 0.0001 (****). See also Figure S5.

We next sought to define the cis-acting elements required for local translation of the short CDS transcripts. However, in marked contrast to the long CDSes, cDNA reporters introduced by lentivirus poorly recapitulate endogenous gene enrichment, even when they contained 5′ and 3′ UTRs. By contrast, when we used a piggyBac transposon system to introduce full endogenous genomic locus, including introns, then the reporter recapitulated the endogenous gene enrichment. In the context of the full endogenous locus, removal of introns caused a significant decrease in mitochondrial enrichment of four endogenous substrate reporters and one synthetic reporter lacking mitochondrial coding sequences. (Figure 4A). Unlike long CDS transcripts, the 3′ UTR was required for the robust local recruitment of short CDS substrates (NDUFB9, COX7A2 and UQCRQ) (Figures 4B, 4C & S5A). Moreover, removing introns and swapping the 3′ UTR with the β-Actin 3′ UTR abolished mitochondrial transcript targeting (Figures 4B, 4C, S5A & S5B).

To identify the minimal elements required for efficient targeting via this pathway, we engineered a synthetic reporter containing only the endogenous UTRs from COX7A2, one of the top hits for locally translated short CDSes, along with a single intron and part of the mCherry protein sequences. The mRNA of this synthetic reporter was efficiently targeted to the OMM, comparable to the endogenous COX7A2 reporter (Figure 4C). Additionally, another synthetic reporter containing the endogenous UTRs from NDUFB9, a single intron, and a short coding sequence lacking any mitochondrial coding sequence was also effectively targeted to the OMM (Figure 4A). These findings indicate that the UTRs from endogenous short CDSes, along with a single intron, are necessary and sufficient for the transcript recruitment of short CDSes.

### Mitochondrial recruitment of short CDSes is uncoupled from protein import

In contrast to long CDS transcripts, a canonical MTS was not required for recruitment (Figure 4B). Moreover, analysis of the previous APEX-seq “puro resistant” data^8^ revealed that recruitment of short CDSes was not dependent on ongoing translation (Figure S5C). Consistently, bioinformatic analysis indicated that short CDS transcripts, especially those less than 100 aa, tended not to have canonical MTSs^36^ (Figure S5D). However, a functional MTS is essential for protein import. Removing the MTS from NDUFB9 reporters did not affect transcript recruitment (Figure 4B) but completely abolished protein import into the mitochondrial matrix, as indicated by a protein complementation assay^37^ using matrix-localized mNeonGreen_2_ (mNG2) _1–10_ and short CDS-mNG2_11_ (Figure 4D). This was also true for the synthetic reporter. Only when a functional MTS from COX5B was added could the short reporter be imported into the mitochondrial matrix (Figure S5E). Taken together, our studies reveal that the rules for localized translation of short CDSes were completely different from long CDSes: for the short CDSes, it was UTRs and presence of intron that were critical whereas the protein coding sequence had a minimal impact.

### Mechanism and physiological role of recruitment of short CDSes to the OMM

**Figure 5.**
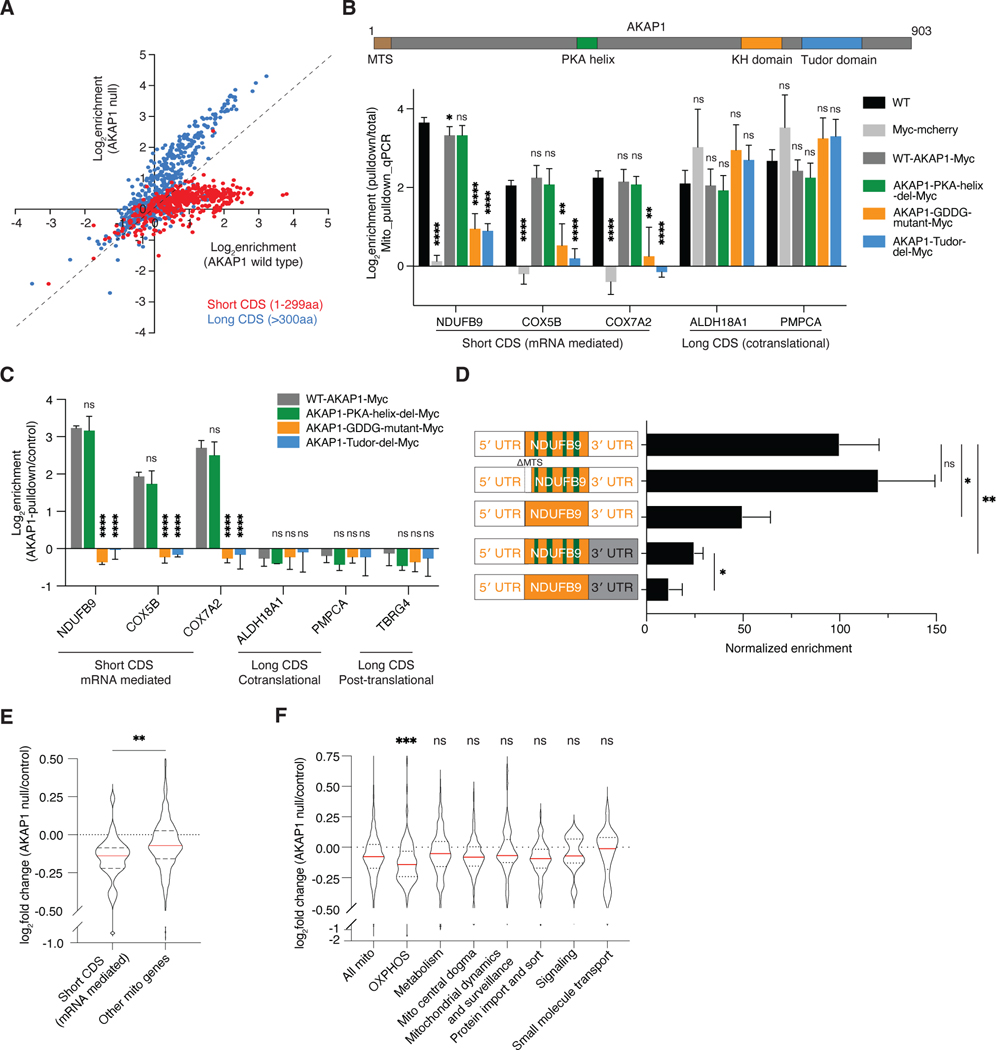
AKAP1 mediates localized translation of short CDSes by specifically binding and recruiting targeted mRNAs. (A) Scatter plot comparing localized translation score (log_2_ enrichment) in monoclonal AKAP1 null cells vs. wild-type using mitochondrial-specific LOCL-TL RNA-seq. CDSes less than 300 aa are in red, while the ones longer than 300 aa are in blue. (B) Bar graph showing enrichment of transcripts at the OMM in wild-type or AKAP1 null cells expressing different Myc-tagged AKAP1 protein variants or Myc-tagged mCherry as a control (N=3). A schematic at the top illustrates different AKAP1 domains. Normalization details are in the Star Methods. *p*-values were generated by comparison to endogenous genes in wild-type cells. (C) Bar graph of enrichment scores for transcripts binding to different Myc-tagged AKAP1 variants, detected by RIP-qPCR (N=3). *p*-values were generated by comparison to endogenous genes in cells expressing wild-type Myc-tagged AKAP1. (D) Bar graph of relative enrichment for various NDUFB9 reporters binding to wild-type Myc-tagged AKAP1, detected by RIP-qPCR (N=3). Values for each reporter are normalized to the positive NDUFB9 reporter (top). (E) Violin plots of the log_2_ fold change of mitochondrial protein abundance in monoclonal AKAP1 null cells compared to wild type, measured by quantitative proteomics. Short CDSes (mRNA mediated) are mitochondrial genes that are locally translated and contain fewer than 200 aa, as shown in Figure 2C. (F) Violin plots showing the log_2_ fold change of mitochondrial protein abundance in different pathways in monoclonal AKAP1 null cells compared to wild type, measured by quantitative proteomics. *p*-values were generated by comparison to all mitochondrial genes. Statistical significance was determined by two-tailed unpaired t-test. *p*-value > 0.05 (ns), *p*-value ≤ 0.05 (*), *p*-value ≤ 0.01 (**), *p*-value ≤ 0.001 (***), *p*-value ≤ 0.0001 (****). See also Figure S6.

We next sought to identify mitochondrial localized RNA binding proteins important for their recruitment. Studies in mammalian cells and drosophila, have identified a few attractive candidates including the La RNA binding protein family member LARP4 and A-Kinase Anchoring Protein 1 (AKAP1)^12,17–19,38–40^, but the details of their roles are not well understood. AKAP1 is a scaffold protein that recruits protein kinase A (PKA), various signaling proteins, and mRNAs to the OMM. It has been proposed that AKAP1 may regulate mitochondrially localized translation through recruitment of LARP4^17,19^. Here we used CRISPR-Cas9 to knock out either AKAP1 or LARP4 proteins to assess their role in mitochondrially localized translation (Figure S6A). Loss of AKAP1 led to a dramatic reduction in localized translation of short CDS transcripts (Figure 5A, Supplemental Table 2) and a roughly 2-fold increase in the enrichment of long CDSes (Figure 5A). However, complete loss of LARP4 did not recapitulate this phenotype, indicating that AKAP1 is able to mediate localized translation of short CDSes in a manner that does not fully depend on LARP4 (Figure S6B, Supplemental Table 2). To functionally dissect the molecular mechanisms of AKAP, we developed a system to rescue. To minimize the effects of AKAP1 overexpression, we used the low-level UBC promoter to express a construct containing wild-type AKAP1 with a Myc tag, which successfully rescued the loss of endogenous AKAP1 (Figure 5B). AKAP1 has four key functional domains: a mitochondrial targeting sequence, a PKA-binding helix, a KH RNA-binding domain, and a Tudor domain that binds RNA-binding proteins^41,42^ (Figure 5B). We generated three AKAP1 variants: one lacking the PKA-binding helix (PKA-helix-del)^17,43^, one lacking the Tudor domain (Tudor-del)^42^, and one carrying a GxxG motif mutation in the KH domain that abolishes RNA-binding function (GDDG mutant)^17,44^. We found that the RNA-binding KH domain and Tudor domain are essential for the mitochondrial recruitment of short CDS transcripts (Figure 5B). This effect was not due to differences in AKAP1 mutant protein expression (Figure S6C). By contrast, the PKA-binding helix, which is involved in PKA signaling, is dispensable for transcript localization.

RIP-qPCR experiments using AKAP1-Myc proteins demonstrated that wild-type AKAP1 specifically and robustly interacts with mRNAs encoding locally translated short CDSes, but not with those encoding locally translated long CDSes (Figure 5C). Critically, the ability of AKAP1 variants to recruit mRNAs precisely correlated with their ability to mediate local translation (Figure 5C). Furthermore, mutations in the short CDS reporter that impaired localized translation, including removal of introns and/or 3′ UTRs also disrupted AKAP1 binding supporting the proposal that AKAP1 mediates short CDS recruitment through direct RNA interactions (Figure 5D).

The ability to specifically prevent localized translation of short CDSes through AKAP1 loss provides an opportunity to explore the physiological consequences of localized translation. Accordingly, we conducted a mass spectrometry-based global proteomics analysis comparing AKAP1 null vs. wild-type cells (Supplemental Table 3). Overall, proteins from the inner mitochondrial membrane and matrix decreased in AKAP1 null cells while the outer mitochondrial membrane and intermembrane space proteins did not change significantly (Figure S6D). Specifically, the abundance of locally translated short proteins significantly decreases in the AKAP1 null cells (Figure 5E). Unlike other mitochondrial pathways, OXPHOS subunits are largely composed of short CDS proteins (Figure S6E) and their protein abundance is also significantly reduced in the AKAP1 null cells (Figure 5F). However, OXPHOS subunits with both long and short CDSes decreased in abundance, potentially due to feedback loops that co-regulate complex subunit abundance^45,46^ (Figure S6F). Proteins regulating translation also decrease significantly, consistent with the fact that mitochondrial ribosomal subunits are also enriched in the short CDS group (Figure S6G).

### The localized translation of long CDS, but not short CDS transcripts, is conserved in yeast

**Figure 6.**
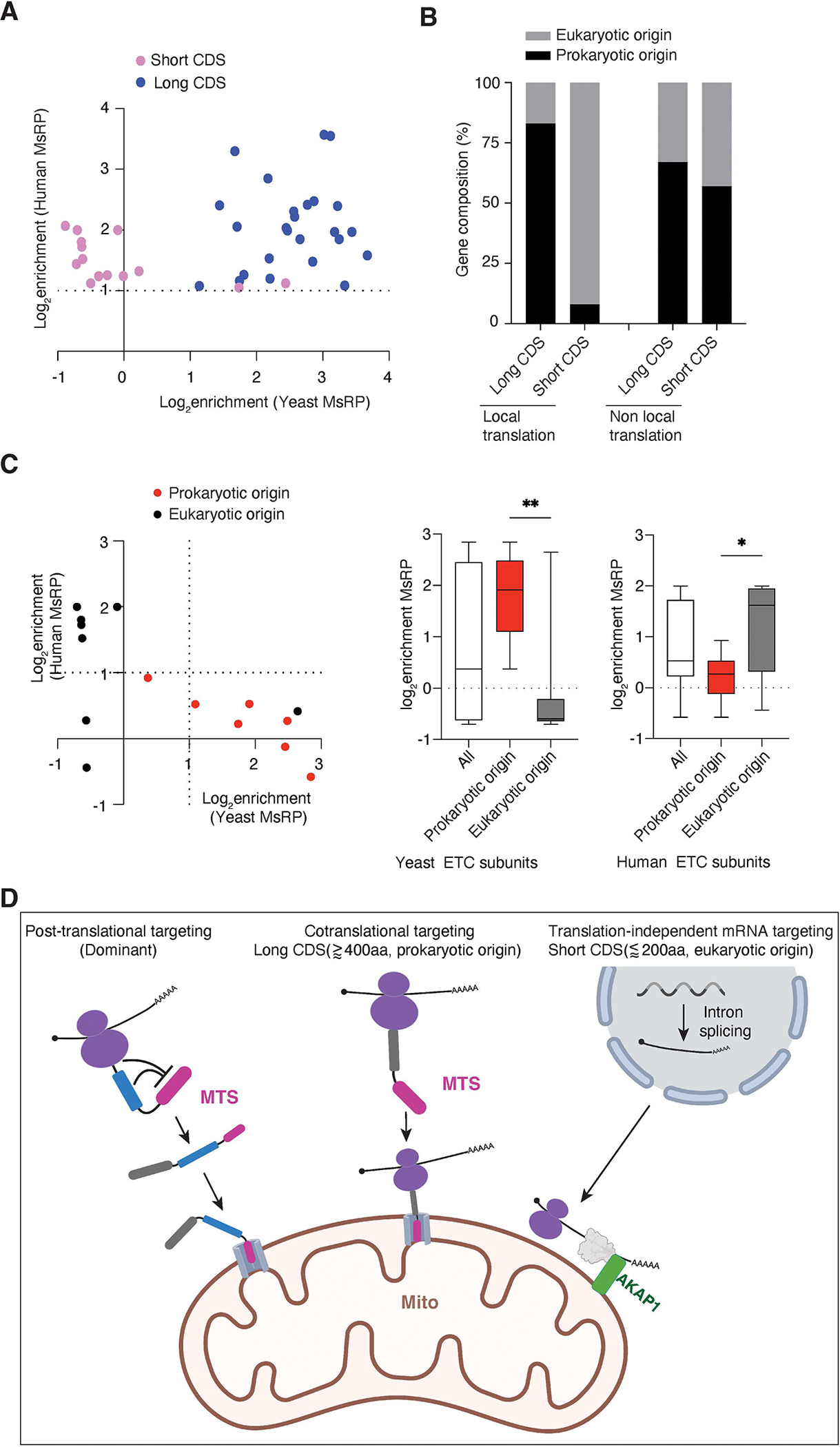
The localized translation of long CDSes, but not short CDS transcripts, is conserved in yeast (A) Comparison of localized translation score for locally translated human mitochondrial genes and their yeast homologs. Colors match Fig. 3B. MsRP stands for mitochondrial-specific ribosome profiling. (B) Bar graphs showing the percentage of prokaryotic vs. eukaryotic origin genes within different groups. (C) Comparison of localized translation scores for conserved ETC homologs. Prokaryotic origin genes are highlighted as red; eukaryotic origin genes are in black or dark gray. MsRP stands for mitochondrial-specific ribosome profiling. *p*-value ≤ 0.05 (*), *p*-value ≤ 0.01 (**). (D) Schematic summary model for three distinct pathways of nuclear-encoded mitochondrial protein import. The majority of proteins are imported post-translationally (left). This is mediated by a bipartite signal involving an N-terminal MTS, which is inhibited by the region immediately downstream of the MTS, preventing protein import until translation is completed. This inhibition may be mediated by chaperones and/or cochaperones that are in close proximity to or associated with ribosomes^49,50^. By contrast, a subset of proteins undergoes localized translation via two distinct pathways: Cotranslational targeting to the OMM (center): Proteins longer than approximately 400 aa are cotranslationally inserted into the OMM. These are mostly matrix proteins of prokaryotic origin. Their localized translation is driven by a bipartite targeting signal, which includes a generic MTS and additional amino acids just prior to engagement (e.g., residues 100–250) that do not inhibit the MTS. Translation-independent mRNA targeting to the OMM (right): Transcripts encoding short coding sequences (approximately less than 200 aa) are targeted to the OMM in a translation-independent manner via intron splicing and UTRs. This process is uncoupled from protein import, which still requires a functional MTS. These short CDS proteins are enriched in ETC subunits and are typically of eukaryotic origin. AKAP1 promotes the localized translation of short CDS transcripts by specifically binding and recruiting the targeted mRNAs.

In order to study the conservation of mitochondrially localized translation, we turned to the previously published yeast mitochondrial-specific ribosome profiling dataset^6^. Among locally translated mammalian mRNAs that have yeast homologs, 70% are also locally translated in yeast, while the remaining 30% are only locally translated in mammals (Figure 6A). Notably, we observed that long proteins are almost invariably locally translated in yeast, whereas short proteins are not (Figure 6A).

Mitochondria originated from a eubacterial ancestor, and more than half of nuclear-encoded genes are bacterial in origin^47^. In yeast, longer proteins that are locally translated are more likely to have originated from bacteria^11,13,48^. Consistent with this, prokaryotic origin genes are also more enriched in locally-translated long CDS genes in our human dataset (Figure 6B). By contrast, short CDSes that are locally translated are enriched in eukaryotic origin genes. Since short CDSes are enriched in respiratory chain complexes, we compared all of the ETC subunits that have a yeast homolog. Interestingly, all of the prokaryotic origin genes are locally translated in yeast but not in humans (Figure 6C). By contrast, all of the locally translated genes in humans are of eukaryotic origin. Thus, AKAP1 dependent recruitment of mRNA to the OMM is focused on eukaryotic-derived components of the ETC.

## DISCUSSION

Here we present a versatile system, termed LOCL-TL, which allows one to systematically monitor translation at defined subcellular compartments in mammalian cells. It uses blue light for temporal control of LOV-BirA activity and therefore allows cells to be grown under physiological conditions while maintaining tight temporal control. LOCL-TL thus provides a generalized tool to precisely monitor the global translational landscape with codon resolution at any subcellular compartment where LOV-BirA can be targeted and, in principle, any organism amenable to genetic manipulations.

Application of LOCL-TL to mitochondria resolves a number of outstanding questions about the nature, mechanism, and extent of local protein synthesis at mammalian mitochondria. Overall, about 20% of nuclear-encoded mitochondrial genes are translated specifically at the OMM (Supplemental Table 4). We further identified two distinct modes of mitochondrially localized translation, which differ not only in the class of proteins they act on, but also in their evolutionary origin and mechanisms (Figure 6D). One apparently ancient pathway has conserved substrates from yeast to humans and is dedicated to the localized translation of a critical subset of long CDSes. We show that in human cells these proteins initiate synthesis in the cytosol, but are then cotranslationally recruited to the OMM. The other, which is not present in yeast, mediates localized translation of short CDSes, primarily constituting electron transport chain complexes and mitochondrial ribosomal subunits. These transcripts are targeted to the OMM before translation initiation through an mRNA-mediated system.

Several lines of evidence indicate that local translation of long CDSes is a true cotranslational event mediated by the nascent chain using an unanticipated bipartite targeting signal. The codon resolution, enabled by ribosome profiling, reveals that the transition of protein synthesis from the cytosol to mitochondria occurs approximately between amino acids 250–400, regardless of protein length. The presence of an N-terminal MTS is required for recruitment, but does not distinguish between cotranslational and post-translational substrates. By contrast, cis-element analysis identified a critical region (residues ~100–250) immediately downstream of the MTS and just upstream of the point of engagement that determines whether targeting occurs cotranslationally. Further analysis of this critical region argues for a dominant inhibitory mechanism for post-translational proteins in which this region blocks the ability of the MTS to target nascent chains during translation. Replacing this region with unstructured or intrinsically disordered polypeptides enabled cotranslational recruitment in a variety of different contexts including substrates which would otherwise be post-translationally targeted. Conversely, the insertion of 100 amino acids downstream of the MTS from a post-translationally targeted protein is able to prevent cotranslational targeting. An important open question is the nature of this inhibitory mechanism, which perhaps involves chaperones or co-chaperones that have recently been implicated in mitochondrial targeting during translation^49,50^. This inhibition could also potentially shield nascent chain MTSs from SIFI-mediated cytosolic quality control which degrades unshielded MTS containing proteins in the cytosol^51^.

Why might the cell ensure that a subset of long CDSes is cotranslationally inserted into mitochondria while the remainder use post-translational routes? This strategy could provide priority to a subset of mRNAs for cotranslational insertion without overly taxing the translocon machinery. Yeast studies indicate that cotranslational import is slower than post-translational import due to limits imposed on the rate of cotranslational import arising from the relatively slow speed of protein synthesis (i.e. with cotranslational import, the translocon is occupied for the entire time it takes to synthesize the protein)^1^. Given the limited number of translocons at the mitochondrial membranes, cells would thus need to restrict cotranslational import to a subset of mitochondrial proteins to ensure timely import of the entire mitochondrial proteome^1^. Comparisons of our mammalian data with studies of localized mitochondrial translation in yeast^6^ show that the set of long CDSes that is locally translated in humans is also locally translated in yeast. Interestingly, mitochondrial genes that are of prokaryotic origin are strongly enriched in this group, consistent with the previous yeast study^11,13^. Another yeast study further correlated the presence of precursor proteins at the OMM to mRNA targeting and found that a subset of inner membrane and matrix proteins of prokaryotic origin, particularly electron transport chain subunits, are locally translated at the OMM, while outer membrane proteins, which are mostly of eukaryotic origin, are not^11,48^. This is consistent with our analysis of yeast mitochondrial-specific profiling data^6^. Together, these considerations suggest that localized translation is an ancient mechanism to prioritize the proper production of proteins of prokaryotic origin and import them back to the mitochondrial matrix.

In contrast to the strategy used for the long CDSes, local translation of short CDSes is driven by transcript recruitment, and is uncoupled from protein import. The local recruitment of short CDSes can occur independent of the protein coding sequence but depends on mRNA splicing. Given that splicing occurs in the nucleus, it is surprising that intron splicing plays a role in recruitment to mitochondria. However, a link between nuclear splicing and cytoplasmic mRNA localization has been well established for the Drosophila gene *Oskar* during oogenesis^52,53^ and may play a role in recruiting a subset of mRNAs to centrosomes^54^. We further showed the local recruitment of transcripts encoding short proteins requires the OMM-localized RNA binding protein AKAP1. RIP-qPCR experiments demonstrated that AKAP1 specifically and robustly interacts with locally translated short CDSes, but not the long CDSes or mRNAs encoding cytosolic proteins. Critically, the ability of AKAP1 variants to specifically recruit mRNAs correlated precisely with their ability to mediate local translation. Thus, mitochondrially localized translation of short CDSes is mediated by the ability of AKAP1 to specifically bind and recruit targeted mRNAs. Consistent with the observation that this pathway is not present in yeast, AKAP1 does not have a yeast homolog. Given the significant differences between yeast and human ETC pathways (e.g., yeast lack complex I) and mitochondrial genomes^2^, localized translation of short CDSes may have co-evolved with the mitochondrial genome to coordinate the expression of ETC subunits from nuclear and mitochondrial genomes. AKAP1 is highly expressed in various human cancers and facilitates the metabolic adaptation of cancer cells by upregulating oxidative phosphorylation^41^. Our findings suggest that AKAP1 may promote oxidative phosphorylation by increasing localized translation of ETC subunits, which are highly represented in the small protein group.

More generally, LOCL-TL broadly enables the study of localized translation. Localized translation plays important roles in many biological processes, such as synaptic plasticity, organelle homeostasis, cell migration and embryonic development^55–57^. Dysregulation of mRNA localization and translation has been implicated in various diseases, particularly neurodegenerative disorders, cardiovascular diseases, and cancer progression and metastasis^58^. The LOCL-TL approach should broadly enable the study of where and how local translation is used in these diverse contexts under physiological conditions to ensure the efficient delivery of proteins to their proper location.

## LIMITATIONS OF THE STUDY

Several important questions remain unresolved by our studies. First, by measuring localized translation across pooled mitochondrial populations, our approach may have masked intracellular heterogeneity between individual mitochondria^59^. Investigating this variability could reveal whether localized translation adapts to the distinct energy and biomass needs of individual mitochondrion allowing specialized mitochondrial populations within a cell^59^. Second, we focused solely on nuclear-encoded mitochondrial genes, leaving the role of non-mitochondrial genes translated at the OMM unexplored—these may play roles in regulatory pathways coordinating mitochondrial function with other cellular compartments. Additionally, while we identified AKAP1-mediated mRNA targeting as a key mechanism, the nature of the sequences directing this targeting, as well as the full set of trans factors that mediate recruitment, remains unknown. Finally, the nature of the inhibitory mechanism that prevents post-translational protein import into the mitochondria before translation is completed remains an important open question. Investigating the chaperones or co-chaperones recently implicated in mitochondrial targeting during translation^49,50^ may provide insights into this question.

## RESOURCE AVAILABILITY

### Lead contact

Further information and requests for resources and reagents should be directed to and will be fulfilled by the lead contact, Jonathan S Weissman (weissman@wi.mit.edu).

### Materials availability

Plasmids generated in this study are available on Addgene or upon request. The Addgene information is included in Supplemental Table 5.

### Data and code availability

- All MS proteomics data have been deposited with the ProteomeXchange Consortium^60^ via the PRIDE Inspector Toolsuite^61^ under the accession number PRIDE: PXD054646 and are publicly accessible at https://www.ebi.ac.uk/pride/.
- Sequencing datasets (ribosome profiling and RNA-seq) have been deposited in the Gene Expression Omnibus (GEO) database under accession ID GEO: GSE300977 (https://www.ncbi.nlm.nih.gov/geo/query/acc.cgi?acc=GSE300977).
- We have made use of publicly available software and tools, as stated in the STAR Methods. The metagene plot script has been deposited on GitHub and is publicly accessible at https://github.com/luojingchuan2020/LOCL-TL-analysis, as stated in the key resources table.
- Any additional information required to reanalyze the data reported in this paper is available from the lead contact upon request.

## STAR METHODS

### EXPERIMENTAL MODEL AND STUDY PARTICIPANT DETAILS

HEK293 (ATCC, CRL-1573) and HEK293T (ATCC, CRL-3216) cells were used in this study. They were cultured in DMEM containing 10% fetal bovine serum, 100 units/mL penicillin and 100 μg/mL streptomycin, and 2 mM glutamine at 37°C and 5% CO2. Biotin depletion was achieved by culturing cells in DMEM with 10% charcoal/dextran treated FBS, 100 units/mL penicillin and 100 μg/mL streptomycin, and 2 mM glutamine at 37°C and 5% CO2. For the LOV-BirA experiments, cells were cultured in a dark room incubator for at least four days before induction and were passaged under red light. Prior to the Incucyte growth assays in Figure 1A, HEK293T cells were incubated for two weeks in the indicated media, with nine replicates per group.

### METHOD DETAILS

#### Engineering of LOV-BirA

For cloning, PCR fragments were amplified using Q5 High-Fidelity DNA Polymerase. Vectors were double-digested and ligated with PCR fragments by Gibson assembly, followed by heat shock transformation into competent XL1-Blue bacteria.

To find the optimal LOV domain insertion site, four sites were screened by inserting hLOV1^63^ into wild-type *E.coli* biotin ligase at amino acids 79–82. Constructs were cloned as fusions to Sec61β. The chimeric constructs were transfected into HEK293 cells stably expressing Avi-Rpl10a via lentiviral integration using PEI max. After 16–24 hr, the cells were biotinylated for 10 minutes as described in the “Blue light and biotin induction” section and analyzed by Western blotting. The 80/81 LOV-BirA fusion was selected as it showed the best signal ratio between light and dark conditions. To further improve the ratio, four amino acids prior to the LOV insertion site were deleted and the resulting constructs were tested by Western blotting (Fig. S1C). Similar to LOV-Turbo^22^, removal of these amino acids decreased dark-state activity while maintaining light-state activity; we therefore used this version of LOV-BirA for LOCL-TL. Supplemental Table 5 lists the plasmids generated in this study and their corresponding Addgene IDs.

#### Cell line construction

The C-termini (RPL13, RPL29 and RPL36) and N-terminus (RPL10A) of ribosomal subunits were endogenously tagged via ribonucleoprotein (RNP) delivery of preassembled Cas9 protein (Alt-R^™^ S.p. HiFi Cas9 Nuclease, IDT) with gRNAs and a single-stranded homology-directed repair (HDR) donor from IDT with 150–200 bp homology sequences on the 5′ end and 3′ end of the ATH or HTA tags. ATH stands for AviTag-linker-TEV cleavage site-linker-HA-Tag, while HTA stands for HA-Tag-linker-TEV cleavage site-linker-AviTag. Guide RNAs were designed using CRISPick software^64^. We evaluated two guide RNAs (gRNAs) for each targeted knock-in and screened all resulting clones. For RPL13, the gRNAs used were 5′-AAGAAAAAATAAAGCCCTCC-3′ (gRNA1) and 5′-GAAAAAATAAAGCCCTCCTG-3′ (gRNA2). For RPL29, the sequences were 5′-CTACTCTGAAGCCTTTGTAG-3′ (gRNA1) and 5′-ATCTACTCTGAAGCCTTTGT-3′ (gRNA2). Lastly, for RPL36, we used 5′-TCAGGGAGAGGGCAGGGGAG-3′ (gRNA1) and 5′-GGGAGGGGCTCAGTCTTTCT-3′ (gRNA2). The gRNA sites in the HDR donor were mutated to prevent Cas9 cutting. The nucleofection procedures were carried out based on published work^65^, except ~30 μM HDR enhancer from IDT was also used immediately after nucleofection to enhance the efficiency of homologous recombination. HEK293T cells were nucleofected with the Amaxa SF cell line kit, using the nucleofection program CM-130, following the manufacturer′s protocols (V4XC-2024, Lonza). Monoclonal cell lines were isolated via single-cell FACS sorting and validated by Western blotting against the HA tag.

Different LOV-BirA fusion proteins, driven by the EF1α promoter (except for ER-localized LOV-BirA which was under the SFFV promoter), were delivered via lentiviral infection at a low multiplicity of infection (MOI of 0.3–0.5). ER-localized LOV-BirA was expressed as a 3X FLAG-LOV-BirA-Sec61β fusion (pJL95), with mCherry and P2A sequences before the fusion protein, and mitochondrially localized LOV-BirA was expressed as 3X FLAG-LOV-BirA-GFP-OMP25TMD fusion (pJL101). We also generated two mitochondrially localized LOV-BirA constructs: 1) a 3x FLAG-LOV-BirA-GFP-TOM5 fusion, and 2) a TOM70-xten16-GFP-LOV-BirA-3x FLAG fusion. Lentivirally transduced cells were FACS sorted based on fluorescent protein expression to generate stable polyclonal cell lines.

The coding sequence of a synthetic 49-amino-acid short-CDS reporter (pJL296) contains a partial BFP sequence, a Myc tag, and an mNG2_11_ sequence; its counterpart (pJL300) is identical except for the addition of an intron.

#### Generation of AKAP1 or LARP4 knockout cell lines and AKAP1 rescue experiments

For the knockout of AKAP1, a gRNA was designed to target the coding strand of exon 1. This gRNA, with the sequence 5′- ACAGACATGAGATTGCGACC-3′, corresponds to amino acid residues Threonine 115 to Proline 121 (T115–P121) of the AKAP1 protein. For the knockout of LARP4, a gRNA was designed to target the non-coding strand of exon 5. This gRNA, with the sequence 5′- AACACTTCAAGAATTAGATC -3′, corresponds to amino acid residues Aspartic Acid 171 to Leucine 177 (D171-L177) of the LARP4 protein. Lentiviral particles containing the guide RNA constructs were produced by transfecting HEK293T cells with the corresponding plasmids using Lipofectamine 3000 (Life Technologies, L3000015). The resulting viral supernatant was then used to transduce the target Cas9-expressing HEK293T cells. Two Days post-transduction, the cells were selected with 1 μg/mL puromycin. Surviving cells were then single-cell sorted into 96-well plates to establish clonal populations. Finally, these clones were expanded and screened by Western blotting to confirm the absence of both AKAP1 and LARP4 proteins. Constructs expressing Myc-tagged wild-type AKAP1 or its variants under a UBC promoter were introduced into AKAP1-null cell lines via lentiviral transduction. To ensure resistance to Cas9, both the WT and variant constructs contained silent mutations in the gRNA recognition site. Transduction was performed at a low multiplicity of infection (MOI of 0.3–0.5), and mCherry-positive cells were subsequently isolated by cell sorting.

#### Microscopy

Mammalian cells were seeded on μ-Slide 8 Well slides (ibiTreat, Cat.No: 80826) the day before imaging and stained when they reached 30–50% confluency. Cells were stained with 100 nM MitoTracker-Deep Red FM dye (Invitrogen^™^, M46753) and Hoechst (Invitrogen^™^, H3570 1:1000) for 45 minutes before 10 minutes fixation with 4% Paraformaldehyde (PFA) in PHEM buffer (60 mM PIPES, 25 mM HEPES, 10 mM EGTA, 2 mM magnesium chloride hexahydrate, pH 6.9). Cells were washed twice briefly with PHEM buffer and then ProLong^™^ Diamond Antifade Mountant (Invitrogen^™^, P36965) was added. For DAPI staining, cells were permeabilized with 0.1% Triton X-100 in PHEM buffer twice for 5 minutes each, prior to 30-min DAPI staining. The fixed cells were imaged on a Zeiss LSM 710 Laser Scanning Confocal Microscope using a 63x oil-immersion objective.

#### Blue light and biotin induction

To activate LOV-BirA for biotinylation, 50 μM biotin was added to the culture and cells were exposed to a 465 nm wavelength, 14-watt blue light (Amazon, model number 884667106091218) for 10 minutes at 37°C and 5% CO2. If used, 100 μg/mL cycloheximide was added to the media 2 minutes prior to the addition of 50μM biotin and blue light treatment. We observed that post-lysis labeling did not increase when the desalted cell lysate was exposed to white light at room temperature for 1 hour (Fig. S2G). Consequently, the Micrococcal Nuclease digestion step in the ribosome profiling protocol does not require protection from light and can be performed under standard laboratory lighting.

For large-scale sequencing experiments, two 150 mm plates (cell confluency: 70 – 80%) were harvested at the same time, following the published method^15^ (section 1.7b), except that only 700 μL polysome lysis buffer (20 mM Tris pH 7.5, 150 mM NaCl, 5 mM MgCl_2_, 1% Triton X-100, 1 mM DTT, 100 μg/mL CHX, 1 U/mL Apyrase (NEB), 24 U/mL Turbo DNase (Ambion), 20 U/mL SUPERaseIn (Ambion)) was used for two plates. We used 5mL Zeba desalting spin columns. For experiments with no cycloheximide treatment, we induced LOV-BirA with a 1-minute treatment of 50 μM biotin and blue light. For each mitochondrial-specific LOCL-TL experiment, we typically harvested cells from 6–8 150 mm plates, adding more if additional material was needed. The negative controls were not exposed to either blue light or biotin addition, unless otherwise specified.

#### Western blot

Cells growing in each well of a 6-well plate were harvested by rinsing with PBS and lysing in 100μL of 1x RIPA buffer for 10 minutes on ice. The lysate was centrifuged at maximum speed and the supernatant was collected for subsequent assays. The Pierce^™^ BCA Protein Assay was performed to determine protein concentration. If the lysis buffer contained DTT, the DC (detergent compatible) protein assay was used instead. Lysates were denatured in a Laemmli buffer at 95°C for 5 minutes. Samples were loaded with equal amounts of protein onto 4 – 12% Bolt Bis-Tris plus gels and run for 45 minutes at 165V in 1x MOPS buffer. Proteins were then transferred to 0.2 μm nitrocellulose membranes with the Trans-Blot Turbo equipment according to the manufacturer′s instructions, blocked with Odyssey blocking buffer and subsequently probed. The following antibodies were used: a mouse anti-HA antibody (1:5000, Roche 12CA5), a mouse anti-FLAG antibody (1:1000, Sigma Aldrich F1804), a rabbit AKAP1 antibody (1:1000, Cell Signaling Technology 5203T), a rabbit LARP4 antibody (1:1000, courtesy from Richard J. Maraia′s lab), and a mouse anti-β-actin antibody (1:1000, Abcam 8226). Li-COR anti-mouse or anti-rabbit secondary antibodies were then used at a 1:10,000 dilution, depending on the primary antibody. Biotin was detected directly using a streptavidin-Alexa Fluor 680 conjugate (Molecular Probes) at a 1:5,000 dilution in TBST with a 10-minute incubation period. We used the Li-COR (Odyssey) system to visualize the blots.

#### Polysome profiling

For polysome profiling of six samples, 10% and 50% sucrose stock solutions were prepared with a 1x polysome gradient buffer containing 20 mM Tris pH 7.5, 150 mM NaCl, 5 mM MgCl2, 100 μg/mL cycloheximide, and 1 mM DTT. The 10% and 50% solutions were made by dissolving 4.5 g and 22.5 g of ultrapure sucrose, respectively, each in a final volume of 45 mL. Following the Biocomp Gradient Master protocol, these stock solutions were used to generate a 10 – 50% continuous gradient in open-top Polyclear centrifuge tubes (14 × 89 mm; Seton Part No. 7030) using the instrument’s preprogrammed settings; the prepared gradients were then stored at 4°C until use. Subsequently, 1 mL of cell lysate was gently layered on top of each gradient, and the samples were centrifuged in a Beckman Coulter Optima XPN-80 Ultracentrifuge using an SW41 rotor at 35,000 rpm for 3 hours at 4°C. Following centrifugation, the sample tubes were kept at 4°C while a Biocomp Gradient Fractionator, equipped with a Triax flow cell and a Gilson fraction collector, was used to continuously record polysome profile traces and collect 12 fractions from the top to the bottom of each gradient according to the manufacturer’s instructions. The experiments were performed in replicates.

#### alamarBlue assays

Cells were seeded at a density of 2,000 cells per well in 96-well plates, with 15 wells prepared per experimental group. Three wells (triplicates) were measured daily, while edge wells were filled with culture media to minimize evaporation artifacts. Cell proliferation was monitored over 4 days by adding 10% (v/v) of the total culture volume of alamarBlue reagent daily, followed by a 2-hour incubation at 37°C. Absorbance was then measured at 570 nm (reduced alamarBlue signal) and 600 nm (background reference), and metabolic activity was calculated by subtracting the OD_600_ value from OD_570_ value for each well. Results were compared between groups during the logarithmic growth phase, typically 3 – 4 days post-seeding.

#### Streptavidin pulldown of biotinylated ribosomes

The procedure was similar to the published method^15^ (section 1.8) with the following exceptions: 100 μL of MyOne Streptavidin C1 magnetic Dynabeads (Invitrogen) were used per 1 mL of cell lysate (harvested as described in the “Blue light and biotin induction” section). The number of bead washes was also increased to two for buffer B and two for low-salt binding buffer (Buffer C), prior to the pulldown. The washed beads were then incubated with cell lysates on an overhead roller for at least 3 – 4 hr at 4 °C. The number of washes with 1 mL of high-salt wash buffer (Buffer D) was also increased to four.

#### Ribosome profiling library generation

Ribosome footprints from both the streptavidin pulldown and total monosome fractions were purified with the Direct-zol RNA Microprep kit (Zymo, R2062) according to the manufacturer’s protocol. The purified RNA was resuspended in 10 μL of 10 mM Tris (pH 7.0) buffer and resolved on a 15% TBE-urea gel in RNAse-free TBE buffer at 200V for 65min. Approximately 5 μg of RNA from the pulldown fraction was used for each library to ensure a robust preparation. Oligoribonucleotide size standards in neighboring lanes were used to guide the excision of gel slices corresponding to 28 – 34 nt ribosome footprints. The footprints were passively eluted from the gel overnight at 4°C in 400 μL of 0.3 M NaCl.

The eluted ribosome footprints were precipitated using GlycoBlue and 2.5 volumes of ethanol, resuspended in 3.5 μL of nuclease-free water, and dephosphorylated for 1 hour at 37°C in a 5 μL reaction containing 0.5 μL of T4 PNK (NEB) and 0.5uL 10X T4 PNK buffer and 0.5 uL of SUPERase·In RNase inhibitor. This solution was used directly for ligation by adding 0.5 μM 5’ pre-adenylated linkers, 3.5 μL of 50% PEG 8000, 0.5 μL of 10X truncated T4 RNA ligase 2 K227Q buffer (NEB), and 0.5 μL of truncated T4 RNA ligase 2 K227Q. Ligation proceeded for 3 hours at 22°C. To remove unligated linkers, 0.5 μL of RecJ and 0.5 μL of 5’ Deadenylase were added, and the reaction was incubated at 30°C for 45 min. Following this incubation, samples were pooled, purified using an Oligo Clean & Concentrator kit according to the manufacturer’s protocol, and eluted with 26 μL nuclease-free water.

rRNA contaminants were removed from the ligation products using the Ribo-Zero kit for mammalian cells. The rRNA-depleted products were then reverse-transcribed in a 20 μL reaction using SuperScript III (Invitrogen) for 30 minutes at 55°C. Following reverse transcription, the RNA template was hydrolyzed by adding 1/10^th^ the volume of 1 M NaOH and incubating for 20 minutes at 70°C. The resulting cDNA was purified with a Zymo Oligo Clean and Concentrate kit and eluted in 10 μL of nuclease-free water. The cDNA was further purified on a 10% TBE-urea gel, eluted overnight, precipitated, and resuspended in 15 μL of nuclease-free water.

The purified cDNAs were circularized in a 20 μL reaction using CircLigase (Epicentre) for 1 hour at 60°C; the enzyme was then inactivated by heating at 80°C for 10 min, according to the manufacturer’s instructions. Circularized products were amplified by 10 – 14 cycles of PCR in a 20 μL reaction using KAPA HiFi HotStart ReadyMix with a fixed primer (5’-AATGATACGGCGACCACCGAGATCTACACTCTTTCCCTACACGACGCTC- 3’) and Illumina indexing primers (IDT). PCR amplicons were gel-purified on 8% non-denaturing TBE gels (180V, 55min), eluted, precipitated, resuspended in 7.5 μL of 10 mM Tris (pH 8.0) buffer or nuclease-free water. Final libraries were quantified using both a Qubit High Sensitivity DNA kit and the Bioanalyzer High Sensitivity DNA assay (Agilent Technologies) and. The libraries were sequenced on an Illumina NextSeq 500 sequencer using a NextSeq 500/550 High Output Kit v2.5 (75-Cycle) to generate 50 bp single-end reads, following the manufacturer’s instructions.

#### RNA-seq library preparation

RNA from both the pulldown and the total mRNA fractions was purified with the Direct-Zol RNA Microprep kit (Zymo, R2062). RNA-seq libraries were prepared with the KAPA RNA HyperPrep Kit with RiboErase (KAPA BIOSYSTEMS, KR1351, Human/Mouse/Rat). The final libraries were sequenced using 50 bp paired-end reads on an Illumina NovaSeq SP sequencer.

#### qPCR assay and quantification

RNA from both the pulldown and the total mRNA fractions was purified with the Direct-Zol RNA Microprep kit (Zymo, R2062). 1–2 μg of RNA was reverse transcribed into cDNA with the Maxima First Strand cDNA Synthesis Kit for RT-qPCR (Life Technologies, K1671). The resulting 20 μL of cDNA (e.g. from 1 μg RNA) was diluted into 100 μL with 80 μL H2O. A 0.5 μL aliquot of the diluted sample was used as the template for a 10 μL qPCR reaction containing 0.43 μM each of forward and reverse primers with the DyNAmo ColorFlash SYBR Green qPCR Kit (Life Technologies, F416L). The qPCR assays were performed on a QuantStudio 7 Flex Real-Time PCR System in a 384-well format.

For the analyses in Figs. 3E and 3F, reporter enrichment was first normalized to the enrichment of endogenously expressed ALDH18A1. Values for each reporter were then represented as a percentage of the value for the ALDH18A1 reporter (top) containing the endogenous 5′ UTR, coding sequence and endogenous 3′ UTR of ALDH18A1. For the analyses in Figs. 4A, 4B, 4C, S5A and S5B, reporter enrichment was first normalized to the enrichment of the corresponding endogenously expressed genes (NDUFB9, COX7A2, COX5B or UQCRQ). Values for each reporter were then represented as a percentage of the value for its corresponding positive control reporter containing the endogenous genomic locus. For the synthetic short CDS (49aa) reporter (light gray), the reporter enrichment was first normalized to the enrichment of endogenously expressed NDUFB9 and then represented as a percentage of the value for the first reporter containing the endogenous genomic locus of NDUFB9. For the analyses in Figs. 5B, 5C and 5D, the value of endogenously expressed H2AC17 (a non-mitochondrial protein) was used as an internal control for normalization to minimize batch effects. For the analyses in Figs. 5C & 5D, the log_2_ enrichment of each individual gene was calculated by normalizing it to its value in control cells expressing Myc-tagged mCherry proteins. For the analysis in Fig. 5D, values for each reporter were then represented as a percentage of the value for the positive NDUFB9 reporter (top).

#### Split mNeonGreen2 system

To generate cell lines expressing mNG2_1–10_ in the mitochondrial matrix, HEK293T cells were transduced with lentivirus produced from a construct containing mNG2_1–10_ fused to a mitochondrial matrix targeting sequence^66^. Transduced cells were then sorted into 96-well plates as single-cell clones using a Sony Cell Sorter (SH800S) after 72 hr. After expansion, correct cell lines were confirmed by successful complementation upon transfection with a construct expressing mNG2_11_ targeted to the matrix.

#### Flow cytometry

For all reporter experiments, HEK293T cell lines stably expressing mNG2_11_ in the mitochondrial matrix were transduced with lentiviruses containing the indicated reporter constructs. After 48 – 72 hours, the cells were harvested, washed once with ice-cold PBS, and analyzed by flow cytometry. The signal from mNG2 complementation was quantified in cells expressing mCherry, which indicated successful transduction with the reporter construct. All experiments were performed at least twice. Samples were analyzed on either an Attune NXT Flow Cytometer (Thermo Fisher), and the data were processed using FlowJo v10.8.2 software (BD Life Sciences).

#### RIP-qPCR assays

RIP-qPCR assays were conducted according to a previously described protocol^67^, with the exception that Pierce^™^ Anti-c-Myc Magnetic Beads (Thermo Scientific, 88842) were used. After the final wash step, the bead mixture was incubated for 5 minutes at room temperature with three volumes of TRIzol LS. The magnetic beads were then separated using a magnetic rack, and the associated RNAs were purified using the Direct-zol RNA MicroPrep kit (Zymo, R2062) according to the manufacturer’s instructions. RNA concentrations were measured with a Qubit HS RNA Kit to ensure the same RNA input for subsequent qPCR.

#### Seahorse assay

Seahorse assays were performed according to a previously described protocol^68^. For two weeks prior to the experiment, HEK293T cells were cultured in either standard media or biotin-depleted media. One day before the assay, cells from the standard media group were seeded at 50,000 cells per well, while cells from the biotin-depleted group were seeded at 70,000 cells per well into a 96-well Seahorse plate. Each condition was plated in 12 replicate wells, avoiding the plate edges. After the assay, viable cell numbers were quantified with a Countess II automated cell counter (Invitrogen) using trypan blue staining. The resulting Seahorse data were subsequently normalized to the cell number in each well.

#### Total proteome sample preparation

Cells were cultured to 70% confluency and washed with PBS three times. Cells were lysed in urea denaturing buffer (8M urea, 150mM NaCl and 50mM EPPS pH8.0, containing mammalian HALT protease inhibitor cocktail (Sigma), and Phos-STOP) Cell lysates were sonicated on ice for 10 seconds at level 5, and resultant extracts were clarified by centrifugation for 10 minutes at 15,000 × g at 4°C. Lysates were quantified by BCA and ~50 μg of protein was reduced with TCEP (10mM final concentration for 30 minutes) and alkylated with Chloroacetamide (20mM final concentration) for 30 minutes. Proteins were chloroform-methanol precipitated using the protocol in SL-TMT protocol^69^, reconstituted in 200 mM EPPS (pH 8.5), digested by Lys-C for 2 hr at 37°C (1:200 w:w LysC:protein) and then by trypsin overnight at 37°C (1:100 w:w trypsin:protein). ~25 μg of protein was labeled with 62.5 μg of TMTpro for 120 minutes at room temperature. After checking labeling efficiency, samples were quenched with hydroxylamine solution at ~0.3% final concentration (weight in water), pooled, and desalted using C18 solid-phase extraction (SPE) (SepPak, Waters). Pooled samples were offline fractionated with basic reverse phase liquid chromatography (bRP-LC) into a 96-well plate and combined for a total of 24 fractions^70^ before desalting using C18 StageTips (packed with Empore C18; 3M Corporation), and subsequent LC–MS/MS analysis.

#### Liquid chromatography and mass spectrometry data acquisition

Mass spectrometry data were collected using an Orbitrap Eclipse mass spectrometer (Thermo Fisher Scientific, San Jose, CA) coupled with Neo Vanquish liquid chromatograph. Peptides were separated on a 100 μm inner diameter microcapillary column packed with ~35cm of Accucore C18 resin (2.6 μm, 150 Å, Thermo Fisher Scientific). For each analysis, we loaded ~2 μg onto the column. Peptides were separated using a 90 minutes gradient of 5% to 29% acetonitrile in 0.125% formic acid with a flow rate of 300 μL/minute. The scan sequence began with an Orbitrap MS^1^ spectrum with the following parameters: resolution of 60K, scan range of 350–1350, automatic gain control (AGC) target of 100%, maximum injection time “auto,” and centroid spectrum data type. We used a cycle time of 1s for MS^2^ analysis which consisted of HCD high-energy collision dissociation with the following parameters: resolution 50K, AGC 200%, maximum injection time 150ms, isolation window 0.6 Th, normalized collision energy (NCE) 36%, and centroid spectrum data type. Dynamic exclusion was set to automatic. The FAIMS compensation voltages (CV) were −30, −50, and −70V.

### QUANTIFICATION AND STATISTICAL ANALYSIS

#### Footprint sequence alignment and gene enrichments

Sequencing reads were de-multiplexed and stripped of cloning adaptors using FastX Splitter, FastX Clipper, and in-house scripts. Reads shorter than 18 nt were discarded, and UMIs were extracted. The GRCh38.p13 human genome reference with Ensembl annotations was used. Reads were first filtered by mapping to Bowtie indices composed of ribosomal RNAs (rRNAs) and transfer RNAs (tRNAs), using Bowtie 1.1.2.0. Reads were then mapped to the genome using TopHat v2.1.1 with the following parameters: tophat --bowtie1 --read-mismatches 1 -g 64 –no-novel-juncs -T. Only uniquely mapped reads from the final genomic alignment were used for subsequent analysis.

Gene-level counts and RPKM values were calculated using the cs count script from the plastid^71^ with a position file that masks regions to which reads cannot uniquely align with the following parameters: --min_length 27 --max_length 36 --center --nibble 13. One mismatch was allowed for the position file, which was generated with the plastid crossmap script. For RPKM calculations, all reads mapping to any annotated transcript were used for the denominator. Gene-level enrichments were calculated by normalizing the log_2_ ratio of biotinylated footprint density (reads per million) within a gene coding sequence (CDS) to the corresponding density in the matched total input ribosome profiling experiment. Genes were excluded from all analyses if they had fewer than 64 CDS-mapping footprints in the total input sample of a particular experiment. For Fig. 2B, data from mitochondrial-OMP25-TMD-LOV-BirA replicate 1 was used to generate the bar graph. For Figs. 2C–F, mitochondrially localized translated genes were defined as the list of nuclear-encoded mitochondrial genes with a log_2_ enrichment > 1.5 in the mitochondrial-OMP25-TMD-LOV-BirA replicate 1 data (highlighted in bold in the tab ‘mito-LOCL-TL (only mito genes)’ of Supplemental Table 2).

#### Metagene plot generation

Metagene plots in Fig. 2F were generated by separating genes into three categories, based on their log_2_ enrichment score in the mitochondrial-specific LOCL-TL data and their protein lengths as follows: 1) Post-translational targeting (log_2_ enrichment < −0.3, more than 400 amino acids), 2) Long CDS cotranslational targeting (log_2_ enrichment >1.5 from replicate 1, more than 400 amino acids), 3) Short CDS translation-independent mRNA targeting (log_2_ enrichment >1.5 from replicate 1, less than 200 amino acids). Codon footprint counts were derived from replicate 1 of the mitochondrial-specific LOCL-TL ribosome profiling data. Transcripts were required to have an average of ≥ 3 footprints per codon. For each transcript, the log_2_ enrichment at each codon was calculated after the addition of a 0.1 RPM pseudo-count to pulldown and total footprint counts and taking the log_2_ of their quotient. The positional log_2_ enrichment for each codon within the transcript was calculated by taking the average log_2_ enrichment for a 21-codon window, with 10 codons on each side of the given codon. For the first 10 codons, the average was taken of all preceding codons and the 10 subsequent codons. These positional log_2_ enrichment values were then normalized prior to metagene analysis. Normalization was performed by identifying the largest absolute log_2_ enrichment value among the codons within a gene, then dividing each codon’s log_2_ enrichment by this identified value. After grouping genes within a metagene plot, the geometric mean and standard deviation of the log_2_ enrichment were calculated at each codon position across the transcripts for the relevant category of genes, excluding transcripts that terminated before that position. The metagene plot script has been deposited on GitHub and is publicly accessible at https://github.com/luojingchuan2020/LOCL-TL-analysis.

#### Gene ontology analysis

GO term enrichment was performed on the set of long CDS genes (more than 400 amino acids) and short CDS genes (less than 200 amino acids) with a log_2_ enrichment >1.5 from replicate 1 of the mitochondrial-specific LOCL-TL ribosome profiling data. g:Profiler^33^ was used for gene ontology analysis and the results of the statistical enrichment analysis of biological pathways are shown in Fig. 2D.

#### RNA-seq

Raw sequencing reads were aligned to the human genome (GRCh38.99) using STAR 2.7.1a^72^ and quantified using featureCounts 1.6.2^73^. Log_2_ enrichment was calculated as the log_2_ ratio of the biotinylated transcript level (fragments per kilobase of transcript per million fragments mapped) to the corresponding density in the matched total input RNA-seq experiments.

#### Gene categorization

##### a. Secretome annotations

We defined the mammalian secretome as the collection of proteins predicted by Phobius^74^ to have either a signal peptide or a transmembrane domain, excluding known mitochondrial proteins annotated in MitoCarta3.0^29^.

##### b. Mitochondrial annotations

Mitochondrial protein annotations were obbtained from MitoCarta3.0^29^.

#### Comparison of yeast and human mitochondrial homologs

From the Saccharomyces Genome Database (SGD)^75^, we retrieved yeast genes possessing a single human homolog. We then compared our dataset with this yeast dataset^6^, specifically focusing on mRNAs locally translated at the mitochondria in mammalian cells. The phylogenetic origin of genes was defined by the algorithm CLIME (Clustering by Inferred Models of Evolution, https://www.gene-clime.org)^47^.

#### Mass spectrum (TMT Data) analysis

Mass spectra were converted to mzXML and monoisotopic peaks were reassigned with Monocole and a database search was then performed using a Comet-based HT using Proteome Discoverer (v2.3.0.420 – Thermo Fisher Scientific)^76–78^. Database searching included all canonical entries from the Human reference proteome database (UniProt Swiss-Prot – 2019–01; https://ftp.uniprot.org/pub/databases/uniprot/previous_major_releases/release-2019_01/) and sequences of common contaminant proteins. Searches were performed using a 20 ppm precursor ion tolerance, and a 0.02 Da product ion tolerance for ion trap MS/MS. TMT tags on lysine residues, peptide N termini (304.207 Da for TMTpro), and carbamidomethylation of cysteine residues (+57.021 Da) were set as static modifications, while the oxidation of methionine residues (+15.995 Da) were set as a variable modification. PSMs were filtered to a 2% false discovery rate (FDR) using linear discriminant analysis by the Picked FDR method, and proteins were filtered to the target 2% FDR level^77^. For reporter ion quantification, a 0.003 Da window around the theoretical m/z of each reporter ion was scanned, and the most intense m/z was used. Peptides were filtered to include only those peptides with >200 summed signal-to-noise ratio across all TMT channels. An isolation purity of at least 0.5 (50%) in the MS1 isolation window was used for samples analyzed without online real-time searching.

For each protein, the filtered peptide-spectral match TMTpro raw intensities were summed and log_2_ normalized to create protein quantification values (weighted average). Using protein TMT quantifications, TMT channels were normalized to the summed TMT intensities for each TMT channel. Normalized log_2_ protein reporter ion intensities were compared using a Student′s t-test and the resultant *p*-values were corrected using the Benjamini-Hochberg adjustment^79^. After confirming the overall quality of the mass spectrum data (log_2_FC and statistical analysis), we focused our analysis on nuclear-encoded mitochondrial genes annotated by MitoCarta3.0. Information regarding protein length, mitochondrial pathways and Sub-mitoLocalization was also obtained from the annotations of MitoCarta3.0. The mass spectrometry proteomics data have been deposited to the ProteomeXchange Consortium via the PRIDE partner repository with the dataset identifier PXD054646. Project name: Deciphering the logic of mammalian mitochondrially localized translation. Project accession: PXD054646.

## Supplementary Material

1 Figure S1. LOCL-TL optimization, related to Figure 1
(A) Western blot analysis of biotinylated Avi-RPL10A (streptavidin) vs. total Avi-RPL10A (HA antibody) upon induction by various BirA constructs in different media. Avi-RPL10A, introduced by lentiviral transduction, may be gradually lost without continuous selection.
(B) Western blot showing biotinylated Avi-RPL10A (streptavidin) and BirA variants (FLAG antibody). Four LOV insertion sites (after aa 79, 80, 81, 82) in wild-type BirA were tested.
(C) Western blot showing biotinylated Avi-RPL10A (streptavidin) and BirA variants (FLAG antibody). The 80/81 LOV-BirA fusion, with or without a 4aa deletion, was tested in various media.
(D) Histograms of log_2_ enrichment for cytosolic LOV-BirA and ER-LOV-BirA in HEK293T cells with lentivirally integrated Avi-RPL10A fusion proteins. Genes are categorized as secretory (blue), MitoCarta3.0 (red) and other cytosolic (gray).

2 Figure S2. LOCL-TL optimization by endogenous tagging of ribosomal subunits, related to Figure 1
(A) Comparison of log_2_ enrichment from proximity-specific ribosome profiling using ER-wild-type BirA vs. ER-LOV-BirA in HEK293T cells with lentivirally integrated Avi-RPL10A.
(B) Left: Structure of the mammalian ribosome-Sec61 complex^26^ highlighting endogenously tagged ribosomal subunits in different colors. Red arrows indicate free termini of tagged subunits. Right: Table showing efficiency of endogenous tagging ribosomal subunits. HTA: HA Tag-TEV cleavage site-AviTag; ATH: AviTag- TEV cleavage site- HA Tag.
(C) Western blot showing biotinylated (streptavidin) and total (HA antibody) ribosomal subunits from different endogenously tagged cell lines.
(D) Polysome profiling results for wild-type HEK293T cells and endogenously tagged RPL29-Avi HEK293T cells.
(E) AlamarBlue assays at 96 hours showing cell viability across different endogenously tagged ribosome subunit cell lines (wild-type (WT), homozygous knock in (homo), and heterozygous knock in (het)). Endogenously tagged RPL36 lines grew slower than WT, possibly due to Avi tag disruption of an alternative isoform^62^. Homozygous RPL10A knock-in lines grew significantly slower, potentially due to ribosomal function disruption as RPL10A is near the nascent peptide exit tunnel.
(F) Comparison of log_2_ enrichment for ER specific LOCL-TL in HEK293T cells with lentivirally integrated Avi-RPL10A vs. endogenously tagged RPL29-Avi. Genes are categorized as secretome (blue) and other (gray).
(G) Western blot showing post-lysis biotinylation under different conditions. ER-LOV-BirA and Avi-tagged RPL29 were either co-expressed (‘C’) in the same cell or mixed (‘M’) from two cell lines (each expressing only ER-LOV-BirA or Avi-tagged RPL29) before lysis. The signal from the mixed group indicates the level of post-lysis labeling. ‘De-salting’ group: lysates went through desalting columns post-harvest to remove biotin. ‘Post-lysis light’ group: cell lysates were exposed to white light during a 1-hour incubation at 25 °C.
Statistical significance was determined by two-tailed unpaired t-test. *p*-value > 0.05 (ns), *p*-value ≤ 0.05 (*), *p*-value ≤ 0.01 (**), *p*-value ≤ 0.001 (***), *p*-value ≤ 0.0001 (****).

4 Figure S4. Localized translation of long CDSes is mediated by a bipartite signal, related to Figures 3
(A) Codon enrichment plots for GFM2 and MIPEP from mitochondrial-specific LOCL-TL ribosome profiling.
(B) Cis-element analysis with chimeric reporters, measured by qPCR (N=3) and normalized as in Figure 3E. *p*-values compare results to the positive ALDH18A1 reporter (top).
(C) Additional cis-element analysis with chimeric post-translational reporters, measured by qPCR (N=3) and normalized as in Figure 3E. CO: Control (no frameshift, but includes the same base-pair mutation as the frameshift SUPV3L1 reporter for stop codon swaps). FS: Frameshift with stop codon swaps. *p*-values compare results to the control reporter derived from SUPV3L1 (top). Elastin (ELN) and MUCIN 6 (MU) are intrinsically disordered sequences not derived from mitochondrial proteins.
Statistical significance was determined by two-tailed unpaired t-test. *p*-value > 0.05 (ns), *p*-value ≤ 0.05 (*), *p*-value ≤ 0.01 (**), *p*-value ≤ 0.001 (***), *p*-value ≤ 0.0001 (****).

5 Figure S5. Localized translation of short CDSes is mediated by UTRs and intron splicing, and is uncoupled from protein import, related to Figure 4
(A) Cis-element analysis with UQCRQ reporters, measured by qPCR (N=3). Dark gray rectangles represent β-Actin 5′ or 3′ UTRs. Normalization details are in the Star Methods. *p*-values compare results to the positive UQCRQ reporter (top).
(B) Cis-element analysis with COX5B reporters, measured by qPCR (N=3). Dark gray rectangles represent β-Actin 5′ or 3′ UTRs. *p*-values compare results to the positive COX5B reporter (top).
(C) Dot plots showing the log_2_ enrichment of APEX-seq OMM with puromycin treatment^8^ in different gene groups separated by protein length, as in Figure 2C.
(D) Bar graphs showing the percentage of mitochondrial genes with (blue) and without (magenta) canonical MTS, separated by protein length cut-off on the x-axis. The number of genes counted per category is indicated above each bar.
(E) Synthetic-mNG2_11_ reporters, with or without the MTS of COX5B, were assessed in HEK293T cells expressing matrix localized mNG2_1–10_ and analyzed as in Figure 4D.
Statistical significance was determined by two-tailed unpaired t-test. *p*-value > 0.05 (ns), *p*-value ≤ 0.05 (*), *p*-value ≤ 0.01 (**), *p*-value ≤ 0.001 (***), *p*-value ≤ 0.0001 (****).

6 Figure S6. AKAP1 promotes localized translation of short CDSes, related to Figure 5
(A) Western blot against LARP4 and AKAP1 to verify knock-out efficiency in isolated clones. GAPDH served as a loading control. Neg: negative control gRNA.
(B) Scatter plots comparing the localized translation score from Mitochondrial-specific LOCL-TL RNA-seq in monoclonal LARP4 null cells vs. wild-type. Colors match Fig. 5A.
(C) Western blot analysis of AKAP1-Myc variant expression levels in rescued cell lines.
(D) Violin plots showing the log_2_ fold change in abundance of mitochondrial proteins from different mitochondrial sub-compartments: OMM (Outer Mitochondrial Membrane), IMS (Intermembrane Space), IMM (Inner Mitochondrial Membrane). *p*-values compare results to all genes control.
(E) Dot plots showing protein length of genes in different mitochondrial pathways. *p*-values compare results to all mito control.
(F) Violin plots of the log_2_ fold change of OXPHOS subunit protein abundance in monoclonal AKAP1 null cells compared to wild-type from quantitative proteomics. *p*-values compare results to all genes control.
(G) Dot plots showing protein length and log_2_ fold change of protein abundance from quantitative proteomics for genes in different functional groups related to mitochondrial central dogma.
Statistical significance was determined by two-tailed unpaired t-test. *p*-value > 0.05 (ns), *p*-value ≤ 0.05 (*), *p*-value ≤ 0.01 (**), *p*-value ≤ 0.001 (***), *p*-value ≤ 0.0001 (****).

9 Supplemental Table 3: Total proteomics data for AKAP1 null and wild-type HEK293T cells
Related to Figures 5E, 5F, S6D, S6F, and S6G

10 Supplemental Table 4: A summary table for mitochondrial genes
Related to Figures 2, 5A, 5E, 5F, S3J, S6B, S6D, S6F and S6G

7 Supplemental Table 1: ER-specific LOCL-TL data
Related to Figures 1F, 1G, S2A and S2F

8 Supplemental Table 2: Mitochondrial-specific LOCL-TL with ribosome profiling or RNA-seq data
Related to Figures 2, 3B, 5A, S3C, S3I, S3J and S6B

11 Supplemental Table 5: Plasmids Generated in This Study and Their Addgene IDs

3 Figure S3. Global characterization of mitochondrially localized translation revealed by mitochondrial-specific LOCL-TL, related to Figures 2 and 3
(A) Western blot of mitochondrial-targeting LOV-BirA fusion proteins (FLAG antibody). Expected sizes are shown below each lane. L stands for protein ladder.
(B) Confocal images of mitochondria (MitoTracker, magenta) and nuclei (DAPI, blue) in cells expressing LOV-BirA-GFP-TOM5 or TOM70-GFP-LOV-BirA. Scale bar represents 15 μm.
(C) Log_2_ enrichment of representative mitochondrial-specific LOCL-TL with LOV-BirA-GFP-OMP25-TMD. Genes are categorized as mitochondrial (red) and other (gray).
(D) Bar graph showing the percentage of mitochondrial (red), secretome (blue) and other cytosolic (gray) genes whose enrichments exceed the indicated log_2_ threshold. Total gene counts per group are displayed above each bar. Data were obtained from HEK293T cells with lentivirally integrated Avi-RPL10A proteins and various mitochondrial-targeting LOV-BirA fusion proteins.
(E) Bar graph showing the percentage of mitochondrial (red) vs. non-mitochondrial (gray) genes whose enrichments exceed the displayed log_2_ threshold in the published APEX-seq OMM dataset with cycloheximide treatment^8^.
(F) Scatter plot of log_2_ enrichment for non-mitochondrial genes vs. expression levels (reads per kilobase per million). MsRP stands for mitochondrial-specific ribosome profiling, which is a mitochondrial-specific version of the LOCL-TL technique combined with ribosome profiling.
(G) Scatter plot of log_2_ enrichment for cytosolic genes (excluding secretory or mitochondrial genes) from APEX-seq vs. mitochondrial-specific LOCL-TL with ribosome profiling. MsRP stands for mitochondrial-specific ribosome profiling. According to the original APEX-seq paper^8^, the recommended cutoff is 0.75. In our study, we used a log₂(enrichment) of 1.5 as the threshold for robustly localized translation at the mitochondria.
(H) Scatter plots of log_2_ enrichment for mitochondrial (red) vs. non-mitochondrial (gray) genes from APEX-seq OMM vs. mitochondrial-specific LOCL-TL ribosome profiling. MsRP stands for mitochondrial-specific ribosome profiling.
(I) Scatter plot of log_2_ enrichment for mitochondrial-specific LOCL-TL RNA-seq with or without cycloheximide. Colors match Fig. 3B.
(J) Comparison of log_2_ enrichment from mitochondrial-specific LOCL-TL ribosome profiling vs. mitochondrial-specific LOCL-TL RNA-seq. Mitochondrial genes are highlighted in dark red.

## Figures and Tables

**Key resources table:** 

REAGENT or RESOURCE	SOURCE	IDENTIFIER
Antibodies		
Anti-HA	Roche	Cat# 12CA5
Anti-FLAG	Sigma Aldrich	Cat# F1804 RRID:AB_262044
Anti-AKAP1	Cell Signaling Technology	Cat# 5203T
Anti-LARP4	courtesy from Richard J. Maraia’s lab	N/A
Anti-beta actin	mAbcam	Cat#8226
IRDye^®^ 800CW Donkey anti-Mouse IgG Secondary Antibody	LI-COR	P/N 926-32212 RRID: AB_621847
IRDye^®^ 680RD Donkey anti-Rabbit IgG Secondary Antibody	LI-COR	P/N 926-68073 RRID: AB_10954442
Streptavidin, Alexa Fluor 680 conjugate	Life Technologies	Cat#S21378
Bacterial and virus strains		
Stellar^™^ Competent Cells	Takara Bio	Cat#636766
XL1-Blue bacteria	Agilent Technologies Inc	Cat#200249
		
		
		
Biological samples		
		
		
		
		
		
Chemicals, peptides, and recombinant proteins		
Dulbecco’s Modified Eagle’s Medium (DMEM)	Life Technologies	Cat#11965092
Fetal Bovine Serum (FBS)	VWR	Cat#97068-085
Charcoal/dextran treated FBS	Thermo scientific	Cat# SH30068.03
Cycloheximide	Sigma Aldrich	Cat# C4859-1ML
DAPI	Sigma Aldrich	Cat# D8417-5MG
Hoechst 33342	Life Technologies	Cat# H3570
MitoTracker Deep Red FM	Life Technologies	Cat# M22426
Trypsin-EDTA (0.25%)	Life Technologies	Cat# 25200056
Sucrose	Sigma Aldrich	S0389-500G
Alt-R^™^ S.p. HiFi Cas9 Nuclease	IDT	Cat#1081058
Lipofectamine 3000	Life Technologies	L3000015
Charcoal/dextran treated FBS	Thermo Scientific	Cat# SH30068.03 Lot # AZE190136
Penicillin-Streptomycin-Glutamine	Life Technologies	Cat# 10378016
Trizol LS Reagent	Life Technologies	Cat# 10296028
Oligomycin	Sigma	495455-10MG
BAM15	Sigma	SML1760-5MG
Rotenone	Sigma	R8875-1G
Antimycin A	Sigma	A8674-25MG
Trypan Blue Stain (0.4%)	Gibco	15250-061
Alt-R HDR Enhancer V2	IDT	10007910
NuPAGE^™^ MOPS SDS Running Buffer (20X)	Thermo Scientific	NP000102
Chameleon^®^ Duo Pre-stained Protein Ladder	Thermo Fisher Scientific	Li-COR Inc NC0738562
GlycoBlue	Invitrogen	AM9516
T4 PNK	NEB	M0201S
PEG 8000, RNase-free	Promega	V3011
T4 RNA Ligase 2, truncated K227Q	NEB	M0351S
Rec J exonuclease	Lucigen	RJ411250
Circligase	Lucigen	CL4111K
Tris pH 8, 1 M, RNase-free	Ambion	AM9856
Tris pH 7, 1 M, RNase-free	Ambion	AM9851
Tris pH 7.5, 1 M, RNase-free	Invitrogen	15567027
Triton X-100 Surfact-Amps Detergent Solution	Life Technologies	85111
Superscript III reverse transcriptase	Invitrogen	18080093
5’ Deadenylase	NEB	M0331S
Critical commercial assays		
Direct-zol RNA Microprep Kits	Zymo Research	R2062
Gel DNA Recovery Kit (capped columns)	Zymo Research	D4008
Oligo Clean & Concentrator	Zymo Research	D4061
DNA Clean & Concentrator - 5	Zymo Research	D4014
alamarBlue HS Cell Viability Reagent	Life Technologies	A50101
Q5 High-Fidelity 2X Master Mix - 500 rxns	New England Biolabs	M0492L
Zeba Spin Desalting Columns, 7K MWCO, 5 mL	Life Technologies	89892
KAPA HiFi HotStart ReadyMix	Roche Diagnostics	7958935001
Qubit RNA HS Assay Kit	Life Technologies	Q32852
Qubit BR RNA Kit	Life Technologies	Q10210
Seahorse XFe96/XF Pro FluxPak Mini	Agilent Technologies, Inc.	103793-100
DyNAmo ColorFlash SYBR Green qPCR Kit	Life Technologies	F416L
Maxima First Strand cDNA Synthesis Kit for RT- qPCR, with dsDNase	Life Technologies	K1671
Pierce^™^ BCA Protein Assay	Thermo Scientific	23227
*DC* (detergent compatible) protein assay	Bio Rad	5000112
Trans-Blot Turbo RTA Mini 0.2 μm Nitrocellulose Transfer Kit, for 40 blots	Bio Rad	1704270
ODYSSEY BLOCKER PBS 500ML	Thermo Fisher Scientific	NC9877369
Ribo-Zero Gold (Human/Mouse/Rat)	Illumina	MRZG126 (discontinued)
NextSeq 500/550 High Output Kit v2.5 (75 Cycles)	Illumina	20024906
KAPA RNA HyperPrep Kit with RiboErase (Human/Mouse/Rat)	KAPA BIOSYSTEMS	KR1351
Deposited data		
RNA-seq and Ribo-seq data	Gene Expression Omnibus	GSE300977 (https://www.ncbi.nlm.nih.gov/geo/query/acc.cgi?acc=GSE300977)
Mass spectrometry data	The ProteomeXchange Consortium^60^ via the PRIDE Inspector Toolsuite^61^	PXD054646
		
		
		
Experimental models: Cell lines		
HEK293T	ATCC	CRL-3216
HEK293	ATCC	CRL-1573 ^™^
		
		
		
Experimental models: Organisms/strains		
		
		
		
		
		
		
Oligonucleotides (5′-3′)		
RPL13 gRNAl	This study	5′-AAGAAAAAATAAAGCCCTCC-3′
RPL13 gRNA2	This study	5′-GAAAAAATAAAGCCCTCCTG-3′
RPL29 gRNA1	This study	5′-CTACTCTGAAGCCTTTGTAG-3′
RPL29 gRNA2	This study	5′-ATCTACTCTGAAGCCTTTGT-3′
RPL36 gRNA1	This study	5′-TCAGGGAGAGGGCAGGGGAG-3′
RPL36 gRNA2	This study	5′-GGGAGGGGCTCAGTCTTTCT-3′
AKAP1 gRNAl	This study	5′-ACAGACATGAGATTGCGACC-3′
LARP4 gRNAl	This study	5′-AACACTTCAAGAATTAGATC-3′
28-mer sizing oligo and cloning control (RNA)	Ingolia et al^16^. McGlincy et al^63^.	5′-AGUCACUUAGCGAUGUACACUGACUGUG/3Phos/
34-mer sizing oligo and cloning control (RNA)	Ingolia et al^16^. McGlincy et al^63^.	5′-AUGUACACGGAGUCGAGCACCCGCAACGCGACUG/3Phos/
RT oligo for sequencing from 5’ end of RPF	Ingolia et al^16^. McGlincy et al^63^.	/5Phos/AGATCGGAAGAGCGTCGTGTAGGGAAAGAG/iSp18/CTGGAGTTCAGACGTGTG-3′
PCR primer - final library	Ingolia et al^16^. McGlincy et al^63^.	5′-AATGATACGGCGACCACCGAGATCTACACTCTTTCCCTACACGACGCTC-3′
Recombinant DNA		
		
		
		
		
		
Software and algorithms		
Metagene plot	This paper	https://doi.org/10.5281/zenodo.16386532
Plastid	Dunn et al.^64^	https://plastid.readthedocs.io/en
*PyMOL*	Schrodinger et al.^65^	v2.5.0
Prism	GraphPad Software	v10.4.1(www.graphpad.com)
bowtie	Langmead et al.^66,67^	v1.1.2.0
Tophat	Trapnell et al.^68^	v2.1.1
STAR	Dobin et al.^69^	v 2.7.1a
python	N/A	v2.7, v3.7 (https://www.python.org)
Other		
465 nm wavelength, 14-Watt Power blue light	Amazon	model number 884667106091218 (discontinued)
Bolt^™^ Bis-Tris Plus Mini Protein Gels, 4–12%, 1.0 mm, WedgeWell^™^ format	Thermo Scientific	NW04122BOX
MyOne streptavidin C1 magnetic DynaBeads	Invitrogen	65002
TBE-urea gel, 15%	Invitrogen	EC68852BOX
TBE-urea gel, 10%	Invitrogen	EC68752BOX
TBE gel, 8%	Invitrogen	EC62152BOX
Pierce^™^ Anti-c-Myc Magnetic Beads	Thermo Scientific	88842

## Highlights

- An optogenetic approach enables monitoring of localized translation in mammalian cells
- Mitochondrially localized translation is mediated by two distinct strategies
- A bipartite signal sequence enables cotranslational translocation of long CDSes
- AKAP1 targets short CDS transcripts to mitochondria independent of translation
