# Supplementary figures and images for: Proximity-specific ribosome profiling reveals the logic of localized mitochondrial translation
Source: Cell. Author manuscript; Available in PMC 2025 Nov 26. (PMC12650760; doi:10.1016/j.cell.2025.08.002)

**A**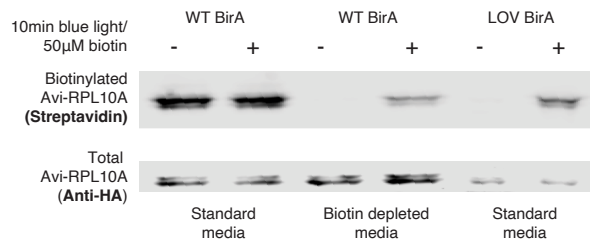**B**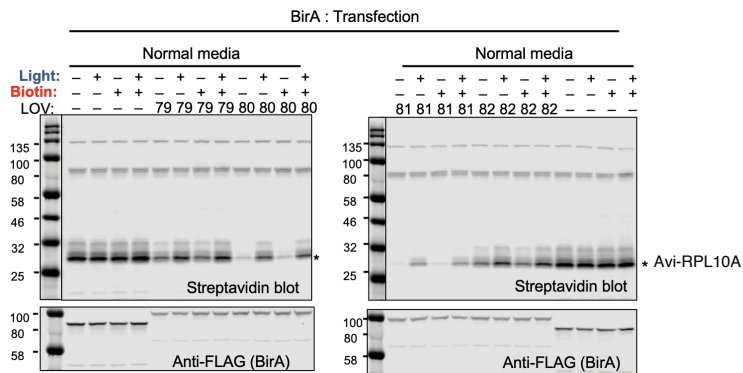**C**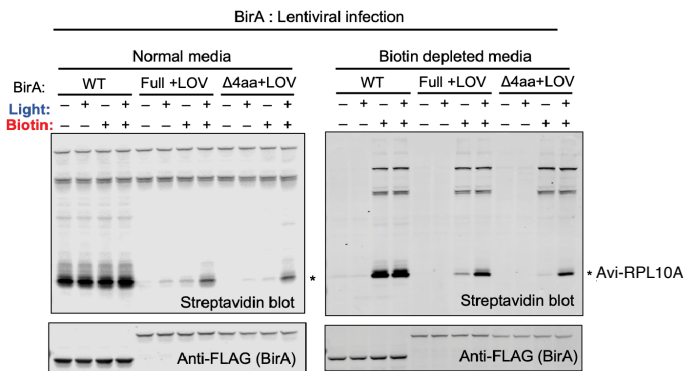**D**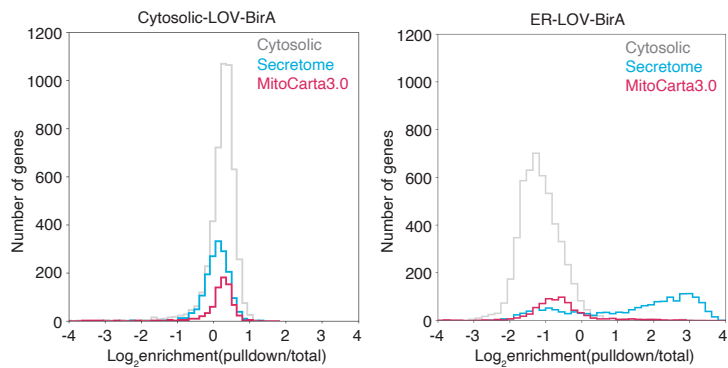

Supplement: 1 — Figure S1. LOCL-TL optimization, related to Figure 1 (A) Western blot analysis of biotinylated Avi-RPL10A (streptavidin) vs. total Avi-RPL10A (HA antibody) upon induction by various BirA constructs in different media. Avi-RPL10A, introduced by lentiviral transduction, may be gradually lost without continuous selection. (B) Western blot showing biotinylated Avi-RPL10A (streptavidin) and BirA variants (FLAG antibody). Four LOV insertion sites (after aa 79, 80, 81, 82) in wild-type BirA were tested. (C) Western blot showing biotinylated Avi-RPL10A (streptavidin) and BirA variants (FLAG antibody). The 80/81 LOV-BirA fusion, with or without a 4aa deletion, was tested in various media. (D) Histograms of log2 enrichment for cytosolic LOV-BirA and ER-LOV-BirA in HEK293T cells with lentivirally integrated Avi-RPL10A fusion proteins. Genes are categorized as secretory (blue), MitoCarta3.0 (red) and other cytosolic (gray). [file NIHMS2102290-supplement-1.pdf]

**A**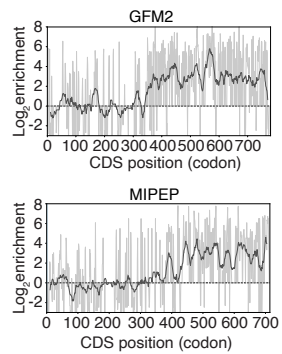**B**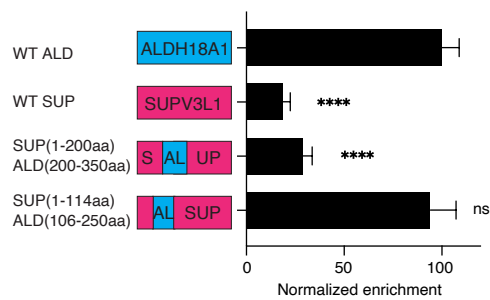**C**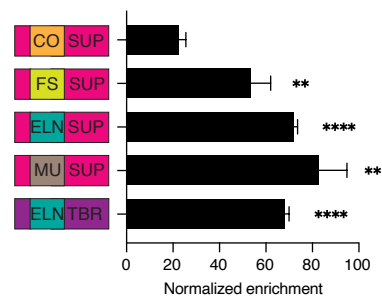

Supplement: 4 — Figure S4. Localized translation of long CDSes is mediated by a bipartite signal, related to Figures 3 (A) Codon enrichment plots for GFM2 and MIPEP from mitochondrial-specific LOCL-TL ribosome profiling. (B) Cis-element analysis with chimeric reporters, measured by qPCR (N=3) and normalized as in Figure 3E. p-values compare results to the positive ALDH18A1 reporter (top). (C) Additional cis-element analysis with chimeric post-translational reporters, measured by qPCR (N=3) and normalized as in Figure 3E. CO: Control (no frameshift, but includes the same base-pair mutation as the frameshift SUPV3L1 reporter for stop codon swaps). FS: Frameshift with stop codon swaps. p-values compare results to the control reporter derived from SUPV3L1 (top). Elastin (ELN) and MUCIN 6 (MU) are intrinsically disordered sequences not derived from mitochondrial proteins. Statistical significance was determined by two-tailed unpaired t-test. p-value > 0.05 (ns), p-value ≤ 0.05 (*), p-value ≤ 0.01 (**), p-value ≤ 0.001 (***), p-value ≤ 0.0001 (****). [file NIHMS2102290-supplement-4.pdf]

**A**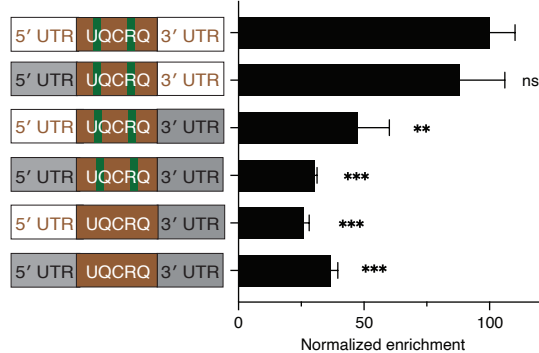**B**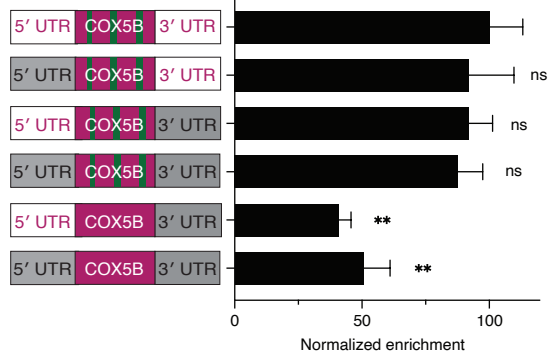**C**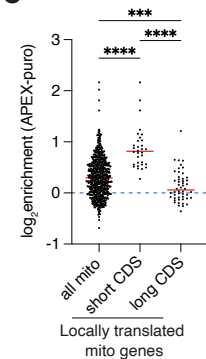**D**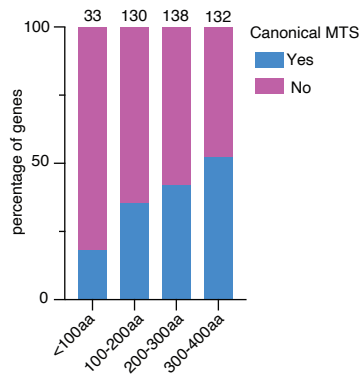**E**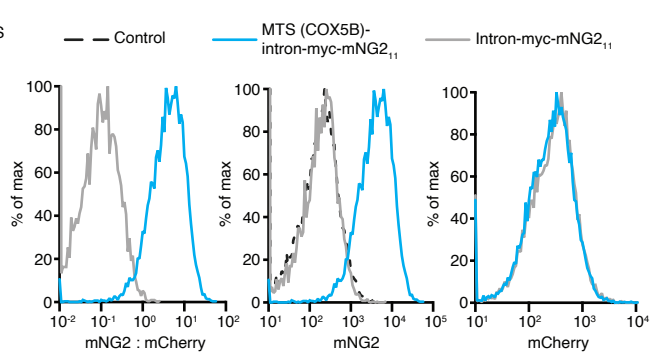

Supplement: 5 — Figure S5. Localized translation of short CDSes is mediated by UTRs and intron splicing, and is uncoupled from protein import, related to Figure 4 (A) Cis-element analysis with UQCRQ reporters, measured by qPCR (N=3). Dark gray rectangles represent β-Actin 5′ or 3′ UTRs. Normalization details are in the Star Methods. p-values compare results to the positive UQCRQ reporter (top). (B) Cis-element analysis with COX5B reporters, measured by qPCR (N=3). Dark gray rectangles represent β-Actin 5′ or 3′ UTRs. p-values compare results to the positive COX5B reporter (top). (C) Dot plots showing the log2 enrichment of APEX-seq OMM with puromycin treatment8 in different gene groups separated by protein length, as in Figure 2C. (D) Bar graphs showing the percentage of mitochondrial genes with (blue) and without (magenta) canonical MTS, separated by protein length cut-off on the x-axis. The number of genes counted per category is indicated above each bar. (E) Synthetic-mNG211 reporters, with or without the MTS of COX5B, were assessed in HEK293T cells expressing matrix localized mNG21–10 and analyzed as in Figure 4D. Statistical significance was determined by two-tailed unpaired t-test. p-value > 0.05 (ns), p-value ≤ 0.05 (*), p-value ≤ 0.01 (**), p-value ≤ 0.001 (***), p-value ≤ 0.0001 (****). [file NIHMS2102290-supplement-5.pdf]

**A**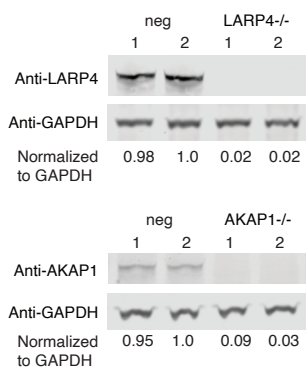**B**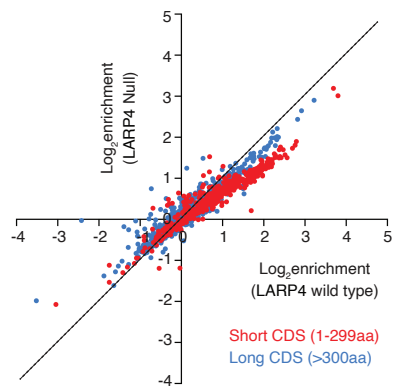**C**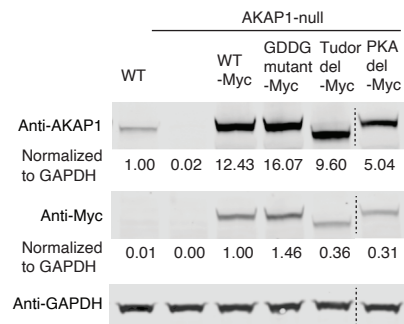**D**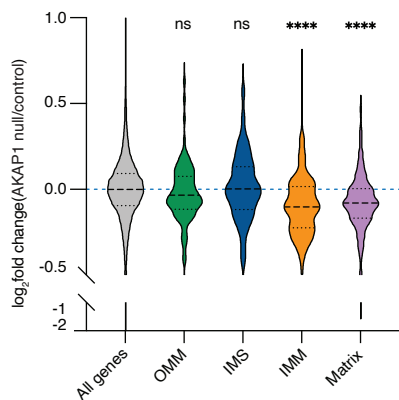**E**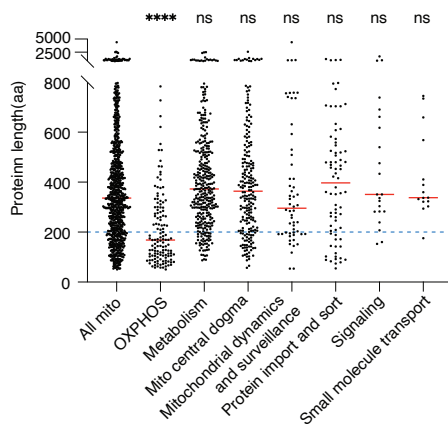**F**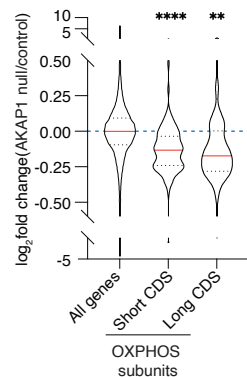**G**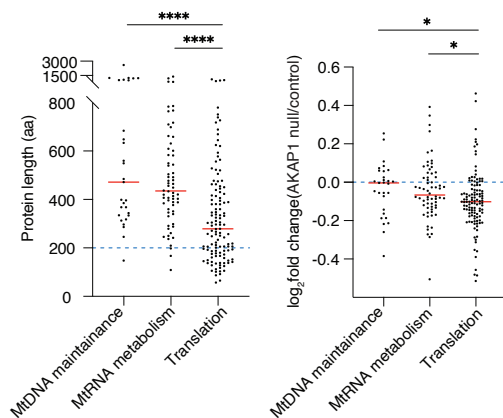

Supplement: 6 — Figure S6. AKAP1 promotes localized translation of short CDSes, related to Figure 5 (A) Western blot against LARP4 and AKAP1 to verify knock-out efficiency in isolated clones. GAPDH served as a loading control. Neg: negative control gRNA. (B) Scatter plots comparing the localized translation score from Mitochondrial-specific LOCL-TL RNA-seq in monoclonal LARP4 null cells vs. wild-type. Colors match Fig. 5A. (C) Western blot analysis of AKAP1-Myc variant expression levels in rescued cell lines. (D) Violin plots showing the log2 fold change in abundance of mitochondrial proteins from different mitochondrial sub-compartments: OMM (Outer Mitochondrial Membrane), IMS (Intermembrane Space), IMM (Inner Mitochondrial Membrane). p-values compare results to all genes control. (E) Dot plots showing protein length of genes in different mitochondrial pathways. p-values compare results to all mito control. (F) Violin plots of the log2 fold change of OXPHOS subunit protein abundance in monoclonal AKAP1 null cells compared to wild-type from quantitative proteomics. p-values compare results to all genes control. (G) Dot plots showing protein length and log2 fold change of protein abundance from quantitative proteomics for genes in different functional groups related to mitochondrial central dogma. Statistical significance was determined by two-tailed unpaired t-test. p-value > 0.05 (ns), p-value ≤ 0.05 (*), p-value ≤ 0.01 (**), p-value ≤ 0.001 (***), p-value ≤ 0.0001 (****). [file NIHMS2102290-supplement-6.pdf]

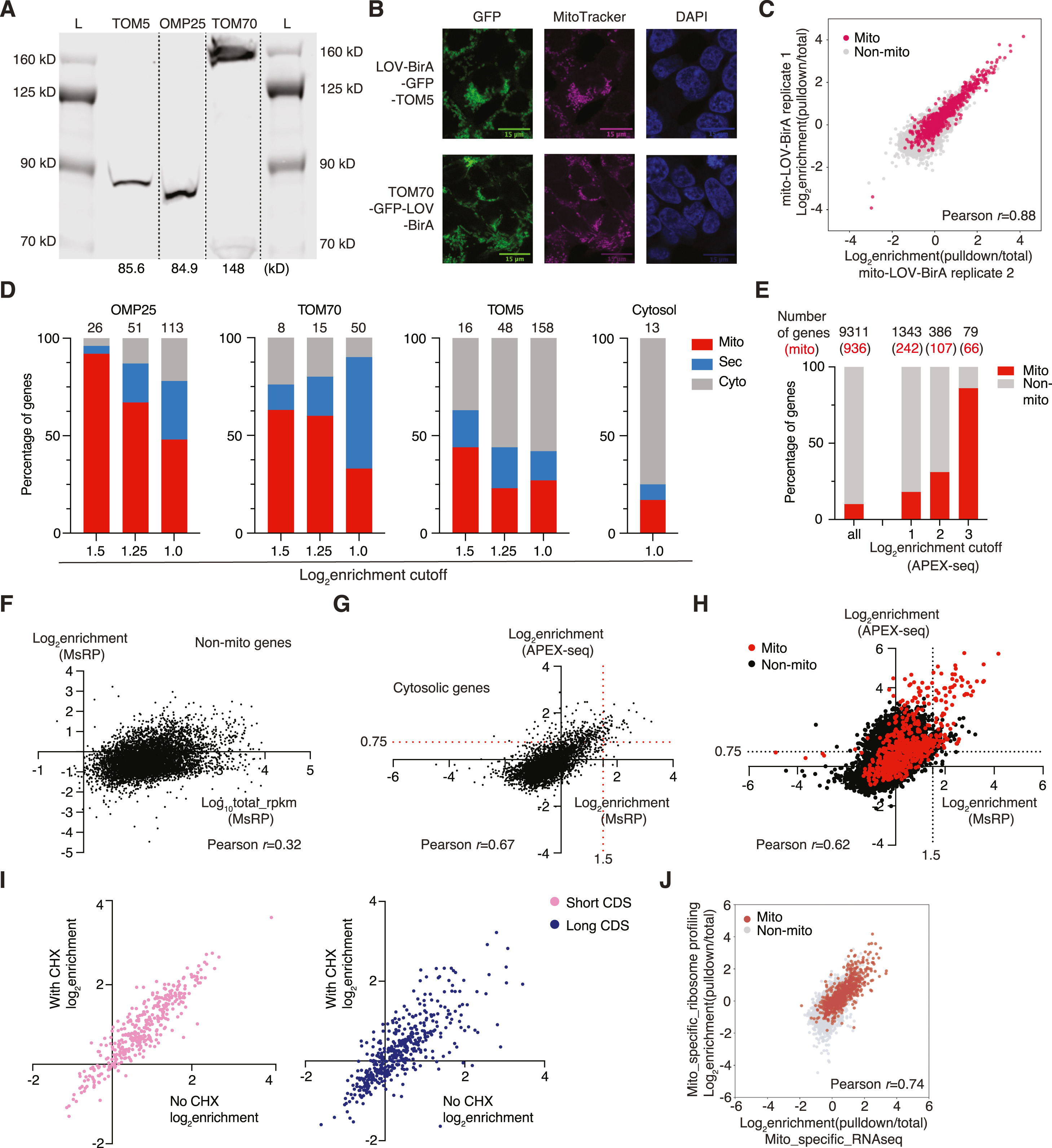

Supplement: 3 — Figure S3. Global characterization of mitochondrially localized translation revealed by mitochondrial-specific LOCL-TL, related to Figures 2 and 3 (A) Western blot of mitochondrial-targeting LOV-BirA fusion proteins (FLAG antibody). Expected sizes are shown below each lane. L stands for protein ladder. (B) Confocal images of mitochondria (MitoTracker, magenta) and nuclei (DAPI, blue) in cells expressing LOV-BirA-GFP-TOM5 or TOM70-GFP-LOV-BirA. Scale bar represents 15 μm. (C) Log2 enrichment of representative mitochondrial-specific LOCL-TL with LOV-BirA-GFP-OMP25-TMD. Genes are categorized as mitochondrial (red) and other (gray). (D) Bar graph showing the percentage of mitochondrial (red), secretome (blue) and other cytosolic (gray) genes whose enrichments exceed the indicated log2 threshold. Total gene counts per group are displayed above each bar. Data were obtained from HEK293T cells with lentivirally integrated Avi-RPL10A proteins and various mitochondrial-targeting LOV-BirA fusion proteins. (E) Bar graph showing the percentage of mitochondrial (red) vs. non-mitochondrial (gray) genes whose enrichments exceed the displayed log2 threshold in the published APEX-seq OMM dataset with cycloheximide treatment8. (F) Scatter plot of log2 enrichment for non-mitochondrial genes vs. expression levels (reads per kilobase per million). MsRP stands for mitochondrial-specific ribosome profiling, which is a mitochondrial-specific version of the LOCL-TL technique combined with ribosome profiling. (G) Scatter plot of log2 enrichment for cytosolic genes (excluding secretory or mitochondrial genes) from APEX-seq vs. mitochondrial-specific LOCL-TL with ribosome profiling. MsRP stands for mitochondrial-specific ribosome profiling. According to the original APEX-seq paper8, the recommended cutoff is 0.75. In our study, we used a log₂(enrichment) of 1.5 as the threshold for robustly localized translation at the mitochondria. (H) Scatter plots of log2 enrichment for mitochondrial (red) v [file NIHMS2102290-supplement-3.jpg]
