# Supplementary material for: Proximity-specific ribosome profiling reveals the logic of localized mitochondrial translation
Source: Cell. Author manuscript; Available in PMC 2025 Nov 26. (PMC12650760; doi:10.1016/j.cell.2025.08.002)

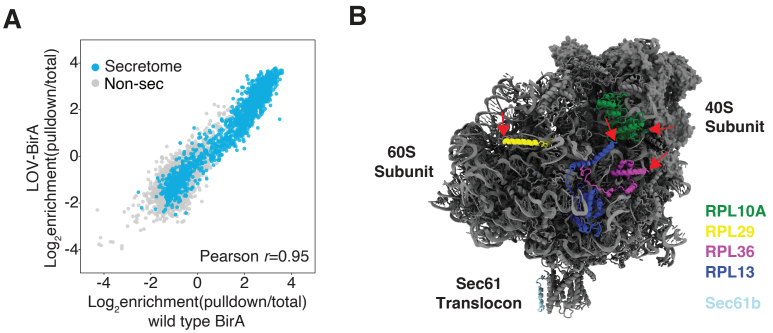

Knock in subunits	Tag	Number of single colony screened	Number of heterozy-gous KI	Number of homozy-gous KI
RPL13	2aa-HTA	185	15 (8.1%)	2 (1.1%)
RPL29	5aa-HTA	96	12 (12.5%)	36 (37.5%)
RPL36	5aa-HTA	284	65 (22.9%)	4 (1.4%)
RPL10A	ATH-2aa	30	0	15 (50%)

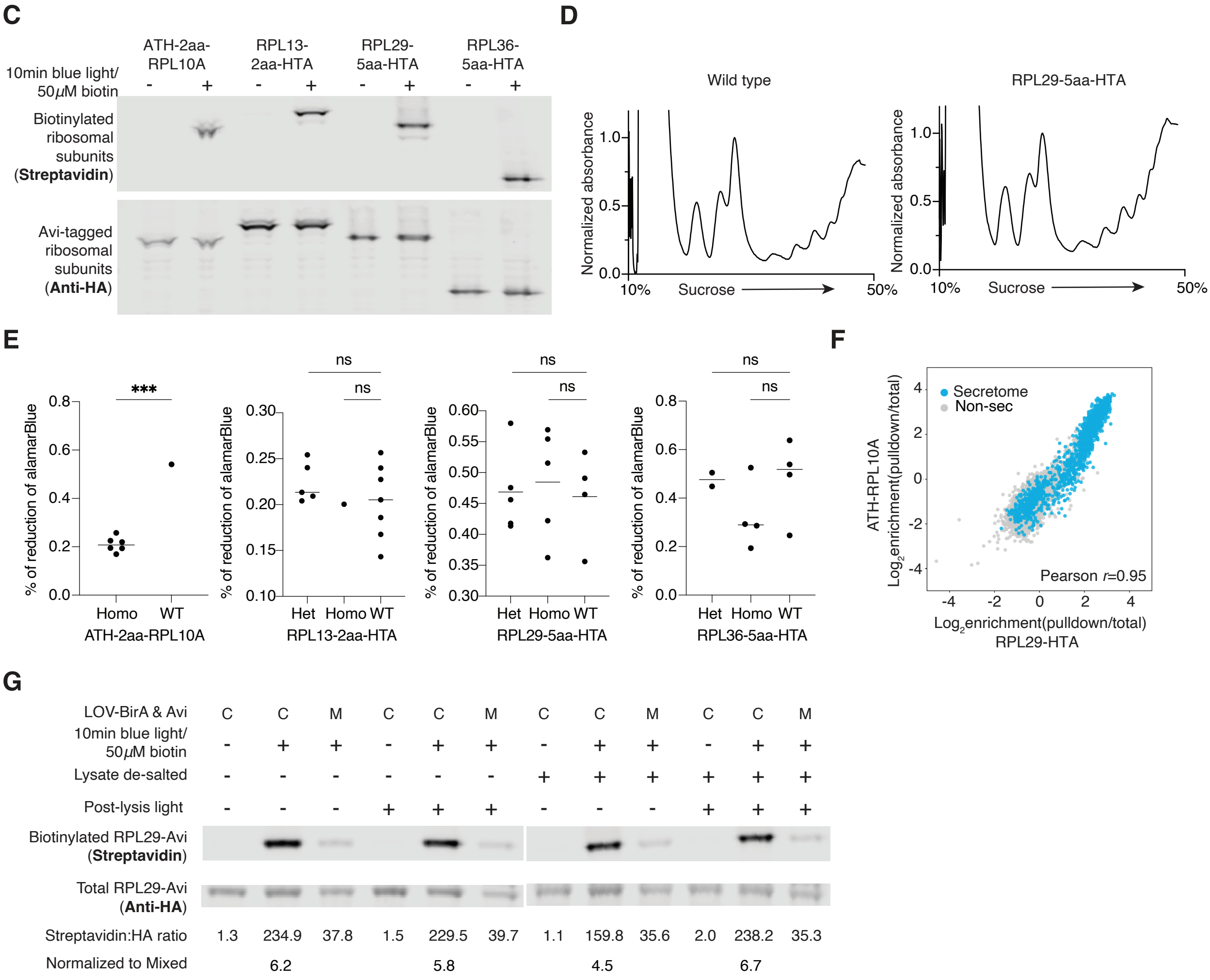

Supplement: 2 — Figure S2. LOCL-TL optimization by endogenous tagging of ribosomal subunits, related to Figure 1 (A) Comparison of log2 enrichment from proximity-specific ribosome profiling using ER-wild-type BirA vs. ER-LOV-BirA in HEK293T cells with lentivirally integrated Avi-RPL10A. (B) Left: Structure of the mammalian ribosome-Sec61 complex26 highlighting endogenously tagged ribosomal subunits in different colors. Red arrows indicate free termini of tagged subunits. Right: Table showing efficiency of endogenous tagging ribosomal subunits. HTA: HA Tag-TEV cleavage site-AviTag; ATH: AviTag- TEV cleavage site- HA Tag. (C) Western blot showing biotinylated (streptavidin) and total (HA antibody) ribosomal subunits from different endogenously tagged cell lines. (D) Polysome profiling results for wild-type HEK293T cells and endogenously tagged RPL29-Avi HEK293T cells. (E) AlamarBlue assays at 96 hours showing cell viability across different endogenously tagged ribosome subunit cell lines (wild-type (WT), homozygous knock in (homo), and heterozygous knock in (het)). Endogenously tagged RPL36 lines grew slower than WT, possibly due to Avi tag disruption of an alternative isoform62. Homozygous RPL10A knock-in lines grew significantly slower, potentially due to ribosomal function disruption as RPL10A is near the nascent peptide exit tunnel. (F) Comparison of log2 enrichment for ER specific LOCL-TL in HEK293T cells with lentivirally integrated Avi-RPL10A vs. endogenously tagged RPL29-Avi. Genes are categorized as secretome (blue) and other (gray). (G) Western blot showing post-lysis biotinylation under different conditions. ER-LOV-BirA and Avi-tagged RPL29 were either co-expressed (‘C’) in the same cell or mixed (‘M’) from two cell lines (each expressing only ER-LOV-BirA or Avi-tagged RPL29) before lysis. The signal from the mixed group indicates the level of post-lysis labeling. ‘De-salting’ group: lysates went through desalting columns post-harvest to remove biotin. ‘Post-lysis light’ g [file NIHMS2102290-supplement-2.pdf]
